# Intravenous administration of IL-12 encoding self-replicating RNA-lipid nanoparticle complex leads to safe and effective antitumor responses

**DOI:** 10.1038/s41598-024-57997-w

**Published:** 2024-03-28

**Authors:** Zihao Wang, Yanni Chen, Hongyue Wu, Min Wang, Li Mao, Xingdong Guo, Jianbo Zhu, Zilan Ye, Xiaoyan Luo, Xiurong Yang, Xueke Liu, Junhao Yang, Zhaolang Sheng, Jaewoo Lee, Zhijun Guo, Yuanqing Liu

**Affiliations:** 1 Immorna (Hangzhou) Biotechnology, Co. Ltd., Hangzhou, 311215 Zhejiang China; 2 Immorna (Shanghai) Biotechnology, Co. Ltd., Shanghai, 201199 China; 3 Immorna Biotherapeutics, Inc., Morrisville, NC 27560 USA

**Keywords:** Tumour immunology, Cancer immunotherapy

## Abstract

Interleukin 12 (IL-12) is a potent immunostimulatory cytokine mainly produced by antigen-presenting cells (e.g., dendritic cells, macrophages) and plays an important role in innate and adaptive immunity against cancers. Therapies that can synergistically modulate innate immunity and stimulate adaptive anti-tumor responses are of great interest for cancer immunotherapy. Here we investigated the lipid nanoparticle-encapsulated self-replicating RNA (srRNA) encoding IL-12 (referred to as JCXH-211) for the treatment of cancers. Both local (intratumoral) and systemic (intravenous) administration of JCXH-211 in tumor-bearing mice induced a high-level expression of IL-12 in tumor tissues, leading to modulation of tumor microenvironment and systemic activation of antitumor immunity. Particularly, JCXH-211 can inhibit the tumor-infiltration of polymorphonuclear myeloid-derived suppressor cells (PMN-MDSCs). When combined with anti-PD1 antibody, it was able to enhance the recruitment of T cells and NK cells into tumors. In multiple mouse solid tumor models, intravenous injection of JCXH-211 not only eradicated large preestablished tumors, but also induced protective immune memory that prevented the growth of rechallenged tumors. Finally, intravenous injection of JCXH-211 did not cause noticeable systemic toxicity in tumor-bearing mice and non-human primates. Thus, our study demonstrated the feasibility of intravenous administration of JCXH-211 for the treatment of advanced cancers.

## Introduction

Interleukin (IL)-12 is a heterodimer (p70) of IL-12A (p35) and IL-12B (p40) and acts as a potent T-helper 1 (Th1) cytokine that promotes host cellular immune responses. IL-12 is primarily produced by activated innate immune cells such as macrophages, monocytes, and dendritic cells and activates T cells and natural killer (NK) cells, leading to increased production of interferon (IFN)-γ and cytotoxic capacity of these cells^1^. Thus, IL-12 has a strong potential to be developed as an antitumor immunotherapeutic agent. However, early phase I and II clinical trials of systemic administrations of recombinant IL-12 protein showed disappointing clinical response rates and severe dose-limiting toxicity^2–5^. Therefore, the development of novel therapeutic modality of IL-12 therapy that has superior therapeutic effects and less toxicity compared to systemic IL-12 protein therapy will greatly benefit patients with advanced cancers.

Self-replicating RNA (srRNA) is a therapeutic and vaccine RNA platform that is derived from an engineered positive-strand RNA viral genome. The srRNA is composed of 5’Cap analog, 5' and 3' untranslated regions (UTRs), poly(A) tail, and multiple coding sequences encoding four viral non-structural proteins (NSPs) and the protein of interest^6^. These NSPs form a complex where RNA-dependent RNA polymerase (RdRp) activity is conferred to initiate the replication of the gene of interest encoded by srRNA. Because of self-replication activity of srRNA, srRNA-based vaccines have been shown to require much lower dose to achieve equivalent vaccine effects against influenza virus compared to conventional mRNA vaccines^7^.

Viral RNA is a potent activator of RNA-sensing pattern recognition receptors (PRRs) such as toll-like receptor (TLR)3, TLR7, TLR8, and retinoic acid-inducible gene I (RIG-I)-like receptors, which induce the expression of type I IFNs, such as IFN-α and IFN-β. Type I IFNs are a potent adjuvant for vaccines against cancer and infection, and potent immune modulators that can convert immunologically ‘cold’ tumors to ‘hot’ tumors^8,9^. Like viral RNA, srRNA can activate multiple RNA-sensing PRRs, leading to production of type I IFNs in human and mouse cells^10^. Presence of activated IFN signaling in tumor microenvironment (TME) has been shown to be positively correlated with a favorable prognosis in multiple types of cancer patients with chemotherapy and immunotherapy^11–13^. Thus, srRNA can provide a great platform for cancer immunotherapy.

Several groups previously demonstrated that intratumoral administration of IL-12 srRNA using nanoparticles eradicated established tumors and inhibited the growth of rechallenged tumors and distal untreated tumors in multiple mouse tumor models including melanoma and colon carcinoma^14,15^. Intratumoral IL-12 therapy has clear advantages over systemic IL-12 therapy, including improving intratumoral concentration of IL-12 and reducing off-target toxicities and adverse effects. However, the disadvantages of intratumoral IL-12 therapy are also obvious, such as the limitation of accessible tumor lesions and the limitation of repeated injection in the same tumor lesion^16^.

In this study, we demonstrate the feasibility of intravenous administration of IL-12 srRNA-lipid nanoparticle (LNP) complex for tumor tissue-tropic expression of IL-12 in tumor-bearing mice. Intravenous injection of IL-12 srRNA-LNP complex induced the expression of IL-12 srRNA predominantly in tumor tissues compared to other organs in tumor-bearing mice. Furthermore, intravenous injection of IL-12 srRNA-LNP complex had comparable antitumor effects to intratumoral injection of IL-12 srRNA-LNP complex in various types of tumor-bearing mice. Finally, intravenous injection of IL-12 srRNA-LNP complex did not cause noticeable systemic toxicity in tumor-bearing mice and non-human primates.

## Results

### Intratumoral treatment with IL-12 srRNA-LNP complex inhibits the tumor growth of syngeneic mice with MC38 mouse colon tumor and humanized mice with patient-derived colon cancer xenograft

**Figure 1:**
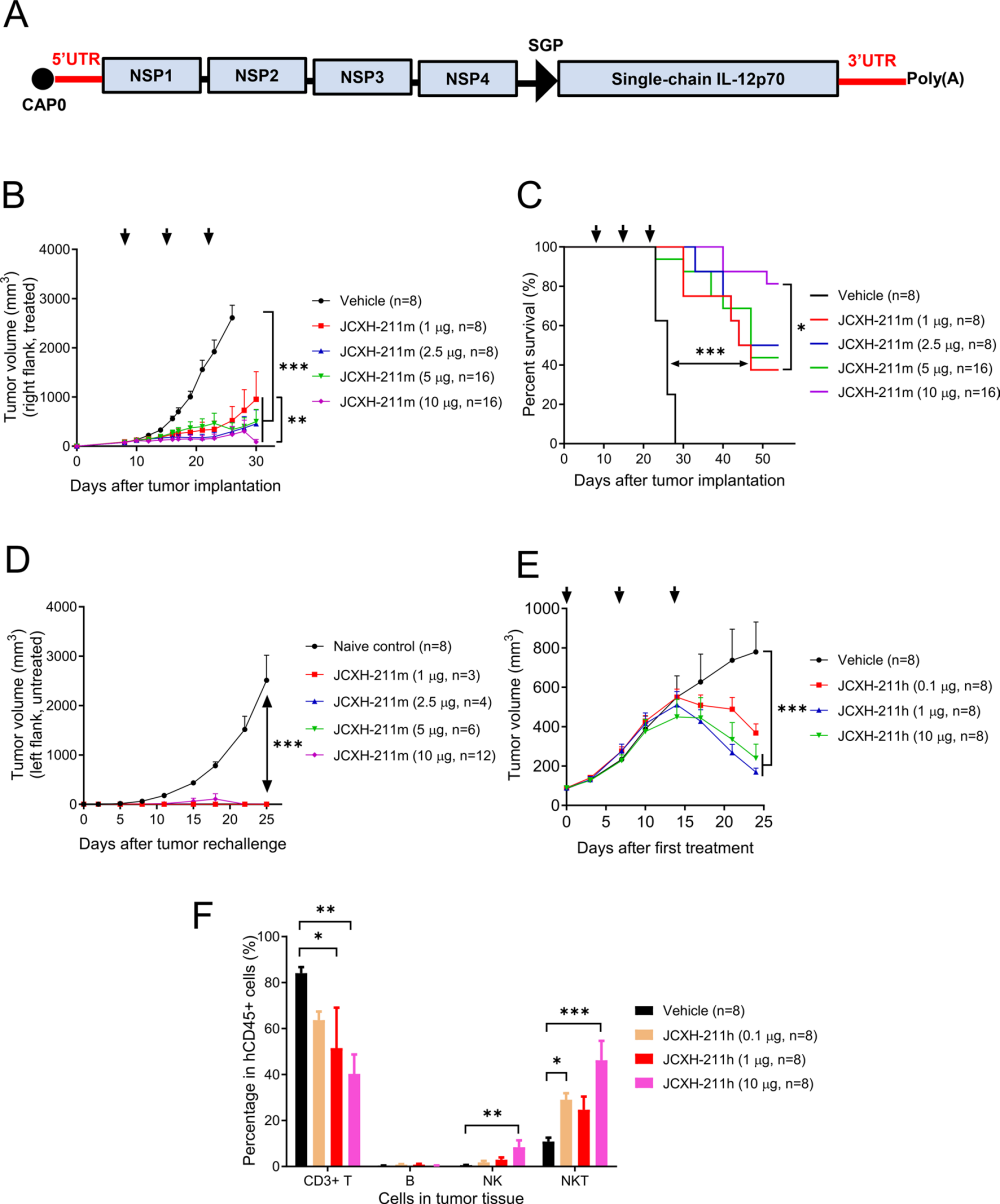
Inhibition of mouse and human colon tumor growth by intratumoral treatment with JCXH-211. (**A**) The srRNA drug substance of JCXH-211 is composed of Cap0 (m7Gppp-), 5’ untranslated region (UTR), alphavirus nonstructural protein (NSP) coding sequences (NSP1-4), subgenomic promoter (SGP), a single-chain IL-12p70 coding sequence, 3’UTR, and poly(A) tail. (**B**,**C**) MC38 tumor-bearing mice were treated intratumorally three times at a 7-day interval with mouse IL-12 srRNA-LNP complex (JCXH-211 m) at indicated doses. (**B**) Tumor volume at indicated time points after first treatment was plotted. (**C**) Survival was plotted using the Kaplan–Meier curve and analyzed using the log-rank test. (**D**) At 46 days after the first treatment, indicated numbers of mice from JCXH-211m-treated groups in (**C**) showed tumor regression and were rechallenged with the same MC38 tumors on contralateral side. No additional treatment was given after tumor rechallenge. The naive control group was a group of naïve mice with MC38 tumor implantation without treatment. (**E**,**F**) Humanized mice with patient-derived xenograft tumors were treated intratumorally once weekly for three weeks with human IL-12 srRNA-LNP complex (JCXH-211h) at indicated doses. (**E**) Tumor volume after the first treatment was plotted. (**F**) At 3 days after last treatment, tumor-infiltrating lymphocytes were analyzed by flow cytometry. The levels of CD3 + CD56- cells (CD3 + T cells), CD19 + cells (B cells), CD56 + CD16 + cells (NK cells), and CD3 + CD56 + CD16 + cells (NKT cells) in human CD45 + cell population of xenograft tumors were plotted. Individual data points in (**B**,**D**–**F**) represent mean ± SEM. The difference in tumor volume and immune cell population among groups were analyzed statistically by (**B**,**D**,**E**) two-way ANOVA with multiple comparison test and (**F**) one-way ANOVA with multiple comparison test. *P < 0.05; **P < 0.01; ***P < 0.001; *ns* not significant (P > 0.05).

Our IL-12 srRNA is derived from an alphavirus, Venezuelan equine encephalitis virus (VEEV) strain TC-83, in the family of positive-strand RNA viruses. This IL-12 srRNA contains Cap 0 analog, TC-83 5’ and 3’ UTRs, poly(A) tail, TC83 NSP 1–4 genes, and single-chain IL-12 gene (Fig. 1A, Supplementary Table 1). Single-chain IL-12 gene encoded IL-12 p40 and p35 subunits fused with a flexible linker, as described previously^17^. To protect srRNA from serum nucleases and improve in vivo delivery of srRNA, the LNP using a proprietary novel ionizable cationic lipid XH-07 was developed to deliver IL-12 srRNA in vivo in our study (Supplementary Table 1). The characteristics of IL-12 srRNA-XH-07 complex, referred to as JCXH-211, were shown in Supplementary Fig. 1A and B. Treatment with JCXH-211 at various doses significantly inhibited the growth of primary MC38 mouse colon tumor and improved the survival of MC38 tumor-bearing mice in a dose-dependent manner compared to control treatment (Fig. 1B,C). Consistent with previous studies of intratumoral IL-12 mRNA therapy^18^ and IL-12 srRNA therapy^14^, intratumoral administration of JCXH-211 could confer full protection of mice from distant tumor rechallenge in the MC38 tumor model (Fig. 1D), suggesting that intratumoral treatment with JCXH-211 induced prophylactic immunity against the same tumors. Furthermore, repeated intratumoral administration of JCXH-211 significantly inhibited tumor growth in human PBMC-reconstituted humanized mice with patient-derived colon cancer xenograft (Fig. 1E). Analysis of tumor-infiltrating lymphocytes (TILs) three days after the last JCXH-211 administration showed significantly increased human NK and NK T cells compared to the vehicle group while the percentage of human CD3^+^ T cells decreased in the JCXH-211-treated groups. The CD3^+^ T cell decrease appeared to be attributed mainly to the CD8^+^ rather than the CD4^+^ T cell fraction (data not shown). It is possible that, JCXH-211 enhanced the activation of both human-against-mouse and anti-tumor responses of grafted T cells in this human PBMC reconstituted NOG mouse model. Such strong T cell activation may promote broad activation-induced T cell death in vivo, resulting in decreased CD8^+^ T cell counts in the tumor (Fig. 1F).

### Systemic injection of JCXH-211 achieves significantly prolonged expression of srRNA in tumor but not normal organs

**Figure 2:**
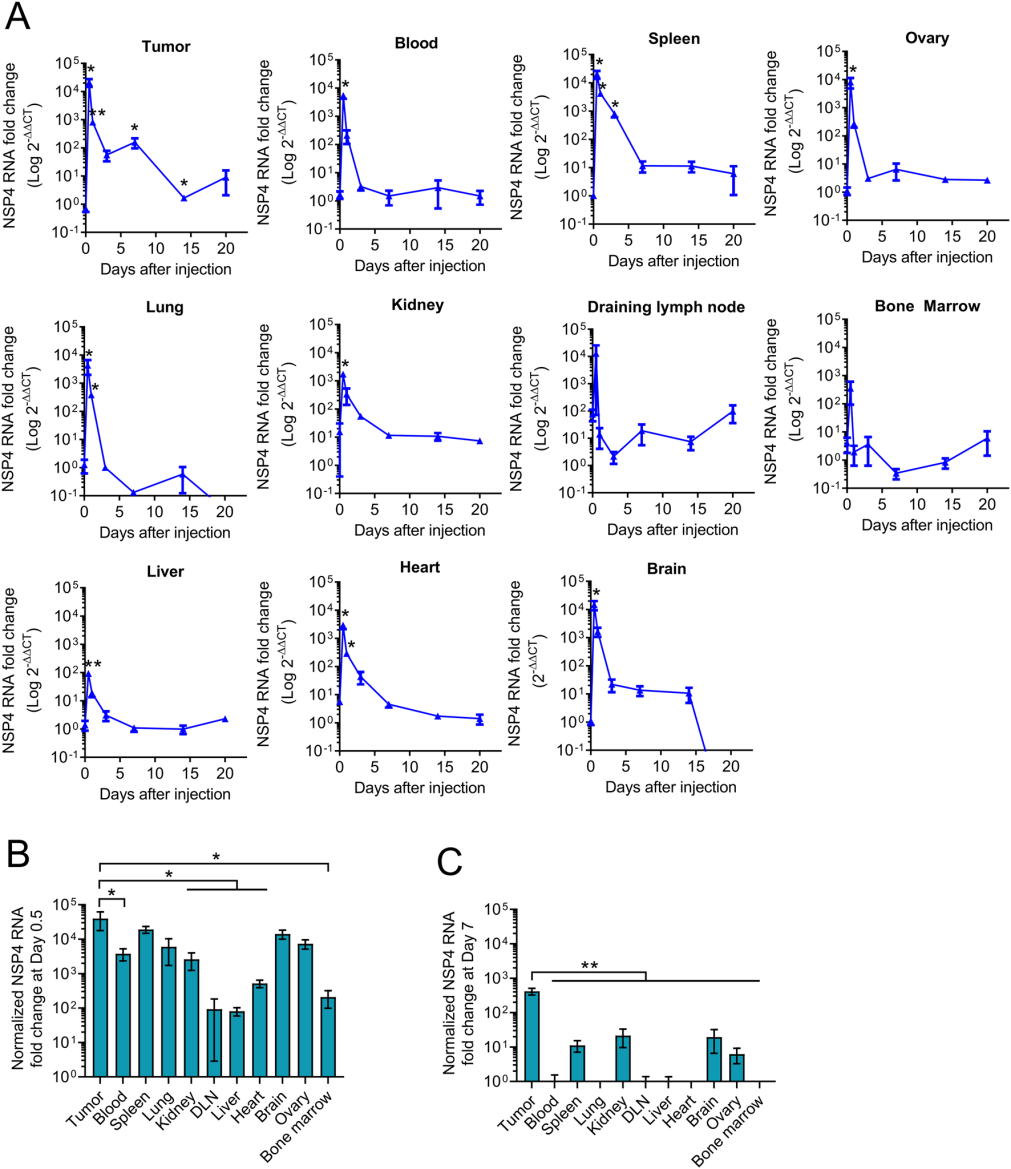
Pharmacokinetics of intravenous injection of JCXH-211. B16F10 tumor-bearing mice were treated once intravenously with JCXH-211m (10 μg). Tumors, blood, spleen, lung, kidney, draining lymph node, liver, heart, brain, ovary, and bone marrow were harvested at indicated time points after treatment. The level of srRNA in these organs was determined by measuring fold changes in NSP4 RNA using RT-qPCR. (**A**) Fold change was represented as follows: 2^−(ΔΔCt) = 2^−[(Ct NSP4 – Ct GAPDH)_JCXH-211m_ − (Ct NSP4 – Ct GAPDH)_control_]. The difference between values at day 0 and day 0.5, 1, 3, 7, 14, and 20 were analyzed statistically by two-tailed t test (n = 3). *P < 0.05; **P < 0.01. (**B**) The fold change values at day 0.5 were normalized to those at day 0. (**C**) The fold change values at day 7 were normalized to those at day 0. The difference among groups were analyzed statistically by one-way ANOVA with multiple comparison test. *P < 0.05; **P < 0.01. Individual data points represent mean ± SEM.

LNP is a critical component of mRNA or srRNA drug products, which facilitates effective protection and delivery of mRNA and srRNA into cells in vivo. Because tumors have rapid but abnormal angiogenesis and dysfunctional lymphatic drainage, LNPs may have increased accumulation and prolonged retention in tumors compared to normal organs when administered intravenously^19,20^. On the other hand, malignant tumors may have an enhanced permissibility for srRNA replication and/or translation due to their immunosuppressive status. Thus, we next asked if systemic injection of JCXH-211 induced comparable antitumor effects to intratumoral injection of JCXH-211. To this end, we first determined the biodistribution of srRNA after intravenous injection of JCXH-211 in tumor-bearing mice. As shown in Fig. 2A, the level of srRNA was significantly elevated in tumor, blood, spleen, lung, kidney, liver, heart, brain, and ovary at 12 h after intravenous injection of JCXH-211, compared to before injection of JCXH-211. The level of srRNA was not significantly changed in the tumor-draining lymph node (DLN) and bone marrow before and after intravenous injection of JCXH-211 although tumor exhibited significantly higher level of srRNA than blood, kidney, DLN, liver, heart, and bone marrow at 12 h after intravenous injection of JCXH-211 (Fig. 2B). Interestingly, at 7 days after intravenous injection of JCXH-211, the level of srRNA in blood, lung, DLN, liver, heart, and bone marrow returned to the level before injection, and the level of srRNA in spleen, kidney, brain, and ovary was also significantly reduced when compared to the peak level at 12 h after injection, although higher than that before injection (Fig. 2A). By contrast, tumor expressed over 200-fold higher level of srRNA than normal organs at 7 days after intravenous injection of JCXH-211 (Fig. 2C).

**Figure 3:**
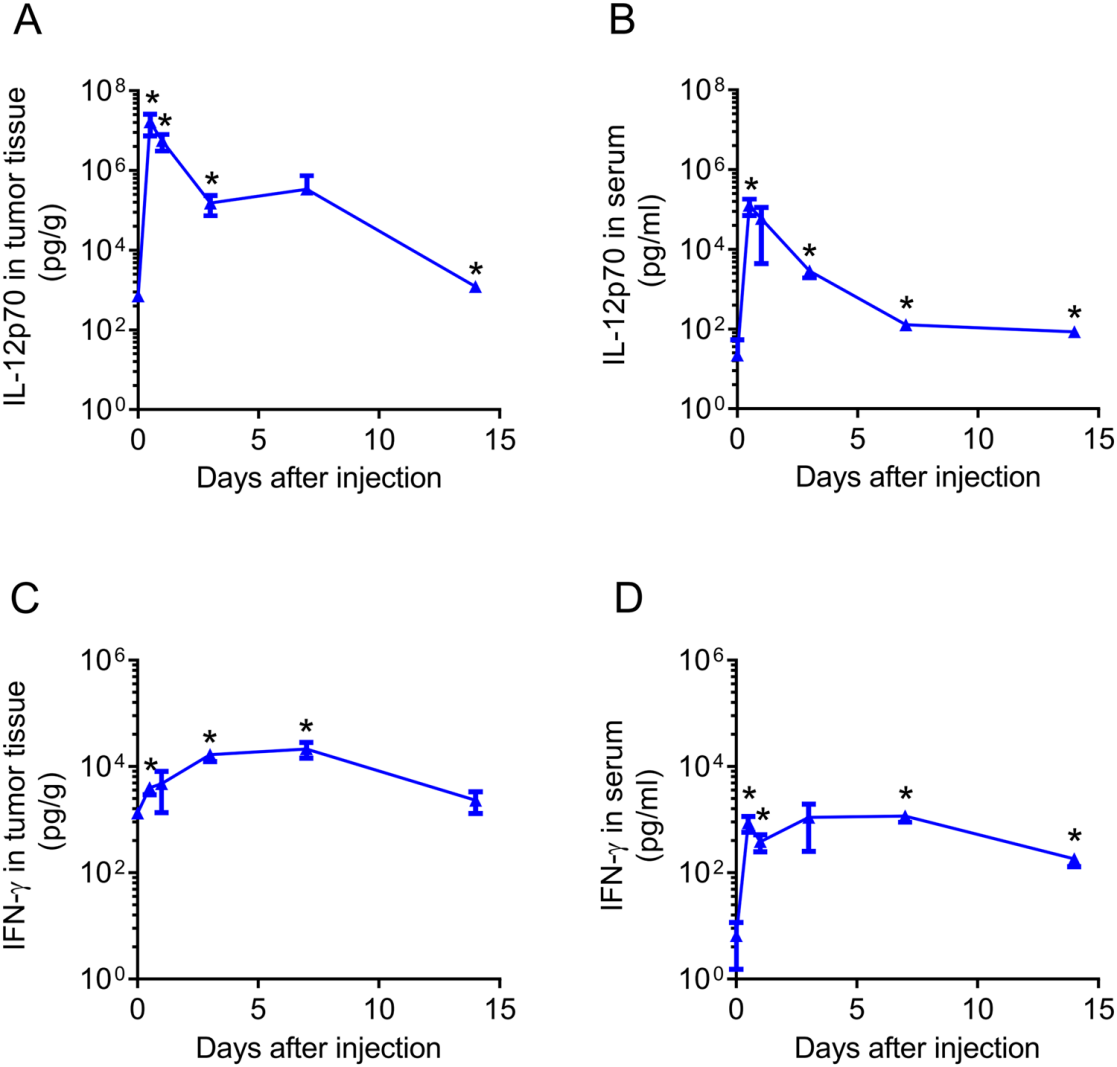
Cytokine production induced by intravenous administration of JCXH-211. B16F10 tumor-bearing mice were treated once intravenously with JCXH-211m (10 μg). (**A**–**D**) Tumors and sera were isolated right before and at 12, 24, 72, 168, and 336 h after JCXH-211 m treatment. The amounts of IL-12p70 and IFN-γ proteins per 1 g tumor weight (**A**,**C**) and 1 ml serum (**B**,**D**) were plotted. The difference in cytokine levels before and at indicated time points was analyzed statistically by two-tailed t test (n = 3). *P < 0.05 vs. before treatment (day 0). Individual data points represent mean ± SEM. See also Supplementary Figs. 2 and 3.

Consistent with the pharmacokinetics and biodistribution of srRNA, IL-12p70 protein exhibited similar pharmacokinetics and biodistribution profiles in tumor-bearing mice with intravenous injection of JCXH-211 (Fig. 3A,B and Supplementary Fig. 2). The level of IL-12p70 protein peaked at 12 h after intravenous injection of JCXH-211 in tumor and blood and was significantly higher for up to 14 days compared to before injection. Mice treated with empty srRNA vector-LNP complex did not lead to increased IL-12p70 in serum, ruling out the possibility that the apparent upregulation was due to endogenous IL-12 gene expression (Supplementary Fig. 3). To determine the downstream effects of IL-12p70 elevation after intravenous injection of JCXH-211 in tumor-bearing mice, we measured the level of IFN-γ, the main effector molecule of IL-12 signaling, in tumor and blood of tumor-bearing immunocompetent mice after intravenous injection of JCXH-211. The level of IFN-γ protein was significantly elevated in both tumor and blood after systemic injection of JCXH-211 and peaked between 3 to 7 days after injection (Fig. 3C,D). If we used 1.06 g/mL as the canonical mouse serum density, the mean IFN-γ concentration in serum at day 7 was about 1098.28 pg/g, while the mean IFN-γ concentration in tumor at day 7 was about 21,338.70 pg/g, so the tumor tissue had over 19-fold higher amounts of IFN-γ than blood at day 7 post injection (P = 0.0077). Our data demonstrated that intravenous administration of JCXH-211 led to prolonged expression of IL-12 srRNA and downstream effector molecules of IL-12 signaling predominantly in the tumor tissue.

**Figure 4:**
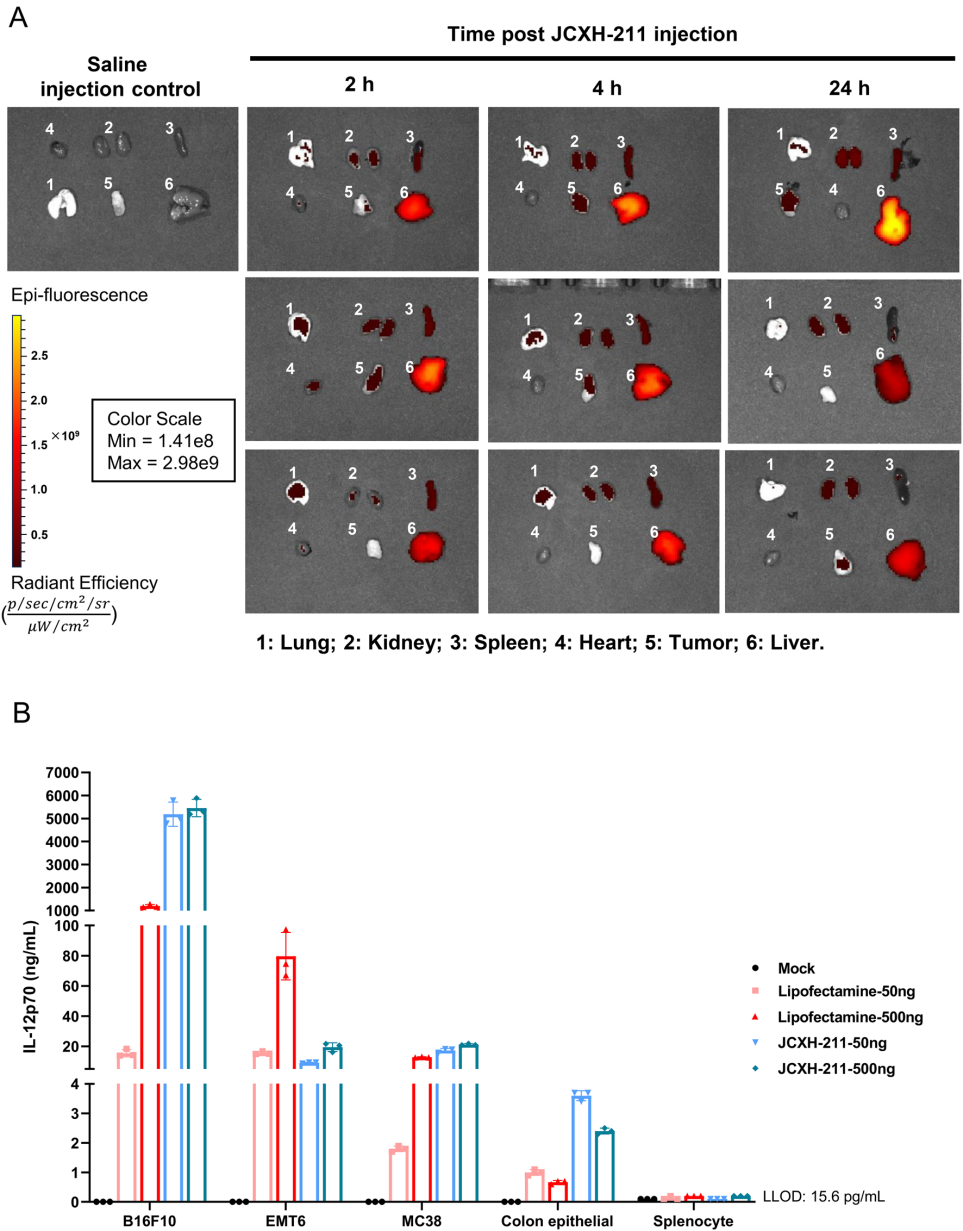
Distribution of LNP-srRNA in mice after intravenous injection and in vitro expression of IL-12p70 by mouse tumor and primary cells after transfection with IL-12 srRNA. (**A**) Female C57BL6 mice were subcutaneously implanted with MC38 cells. 6 days later, Cy5-labeled LNP-srRNA-IL-12 was injected intravenously. The biodistribution of LNP-srRNA in selected organs and tumor was monitored at indicated timepoints with IVIS^®^ Lumina III In Vivo Imaging System (n = 3). Scale bar = 10 mm. (**B**) Murine tumor cells (B16F10, MC38 and EMT6) and mouse primary cells (colon epithelial cells, and splenocytes) were used in this assay. 2 × 10^6^ splenocytes or 8 × 10^4^ other types of cells in 24 well plates were transfected with 50 or 500 ng srRNA encoding mouse IL-12p70 using Lipofectamine MessengerMax or LNP encapsulation (JCXH-211m). Supernatants were collected at 48 h post transfection for IL-12p70 determination by ELISA. Data were presented as mean ± SD, each dot represents one individual sample (n = 3).

To examine the direct biodistribution of the LNP-srRNA formulation in different tissues, mice were intravenously injected with Cyanine 5 (Cy5)-labeled LNP-srRNA-IL-12. Strong fluorescent signals were detected in the liver as soon as 2 h and until 24 h after the injection. The tumor, spleen, kidney, and lung displayed low to medium levels of fluorescent signals while minimal fluorescence was detected in the heart (Fig. 4A). Thus, accumulation of LNP appeared to occur mostly in the liver rather than tumor immediately after intravenous injection. It was plausible that tumor cells were more permissive to srRNA replication and/or translation leading to preferential IL-12 expression. To verify such possibility, an in vitro experiment was conducted in which the murine B16F10, MC38, EMT6 tumor cells, healthy mouse splenocytes and colon epithelial cells were transfected with srRNA encoding IL-12p70 either by lipofectamine or after LNP encapsulation. The content of IL-12p70 was measured in supernatant 48 h after transfection. It was evident that tumor cells were more permissive than healthy primary cells for srRNA-driven IL-12p70 expression. All three tumor cell lines showed IL-12p70 expression although the level varied depending on type of cells with immunologically “cold” B16F10 tumor cells being the most permissive. Neither healthy mouse splenocytes nor primary colon epithelial cells expressed IL-12p70 compared to tumor cells after in vitro transfection (Fig. 4B).

We next asked whether intravenous administration of JCXH-211 would cause systemic toxicity. To answer this question, we determined the level of hepatotoxic biomarker such as aspartate aminotransferase (AST) in the blood of tumor-bearing mice before and after JCXH-211 treatment. The serum AST level was not significantly different between control-treated group and JCXH-211-treated group in B16F10 tumor-bearing mice (Supplementary Fig. 4). Interestingly, we observed that the tumor-bearing mice treated with control treatment gradually elevated the level of serum AST along with increased tumor volume, which is consistent with previous study demonstrating that mice with large B16F10 tumor had elevated levels of hepatotoxic biomarkers in blood, mostly but not exclusively because of metastasis^21^. Surprisingly, B16F10 tumor-bearing mice treated with JCXH-211 showed significantly reduced level of serum AST compared to B16F10 tumor-bearing mice with control treatment (Supplementary Fig. 4). Furthermore, JCXH-211 was administered intravenously into two cynomolgus monkeys (one male and one female) at 100 μg once a week for 3 weeks. Analysis of human IL-12p70 in serum after the first dose of JCXH-211 showed an elevated expression of human IL-12p70 in both animals (Supplementary Table 2). Clinical observation showed no abnormal changes with regards to body weight, food consumption, body temperature, and electrocardiogram parameters during or after JCXH-211 treatment. Blood chemistry found no evidence of hepatic or renal toxicity throughout the study. Only in the male but not female animal, transient elevation in some inflammation indicators such as fibrinogen, plasma-fibrinogen degradation products and C-reactive protein was noted shortly after the second injection. No abnormalities were observed in the gross anatomy of these animals at 7 days after last JCXH-211 administration (Supplementary Tables 3 and 4). Thus, our data suggest that intravenous administration of JCXH-211 would reduce tumor burden without severe systemic toxicity.

### JCXH-211 synergizes with immune checkpoint inhibitors to eradicate B16F10 melanoma resistant to immune checkpoint inhibitor

**Figure 5:**
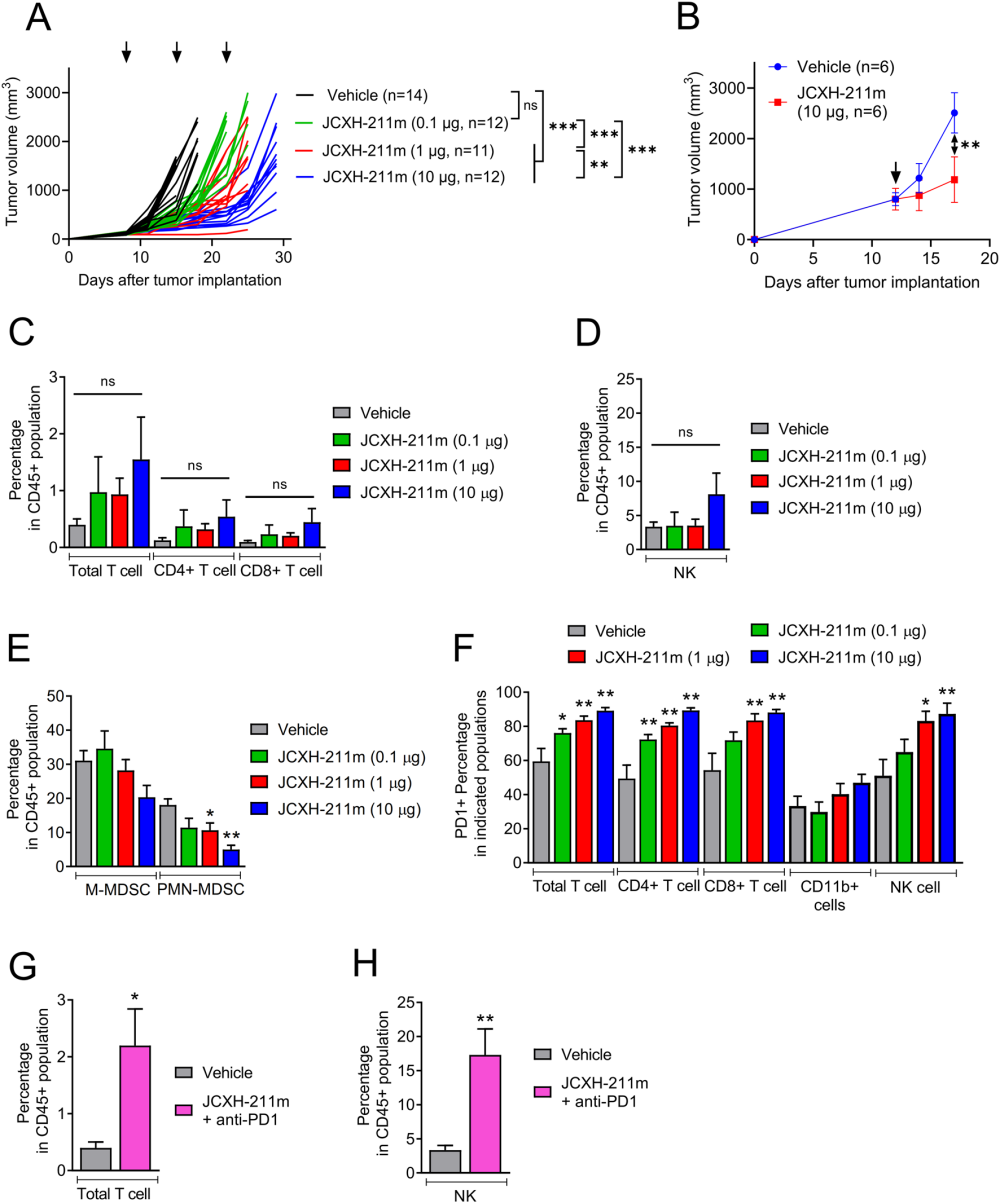
Analysis of tumor-infiltrating immune cells after systemic treatment with JCXH-211. (**A**,**C**–**F**) B16F10 tumor-bearing mice were treated intravenously three times at a 7-day interval with JCXH-211m at indicated doses or PBS control (vehicle). (**A**) The tumor volumes of individual mice were plotted. The difference in tumor volume among groups were analyzed statistically by two-way ANOVA with multiple comparison test. *P < 0.05; **P < 0.01; *ns* not significant. (**B**) Mice bearing a large B16F10 tumor around 800 mm^3^ were treated once intravenously with JCXH-211m at indicated dose or PBS control (vehicle). The aggregated tumor volumes in each group were plotted. The difference in tumor volume among groups were analyzed statistically by two-way ANOVA with multiple comparison test. (**C**–**F**) In the B16F10 tumor-bearing mice shown in panel A, at 3 days after the second treatment, tumor tissues (n = 8) were harvested and analyzed for tumor-infiltrating immune cells, including (**C**) total T cells (CD45 + CD3 +), CD4 T cells (CD45 + CD3 + CD4 +), CD8 T cells (CD45 + CD3 + CD8 +), (**D**) NK cells (CD45 + CD3-CD335/NKp46 +), (**E**) M-MDSC cells (CD45 + CD3-CD11b + Ly6C + Ly6G-), PMN-MDSC (CD45 + CD3-CD11b + Ly6ClowLy6G +). (**F**) PD1 + cells in the population of total T cells, CD4 T cells, CD8 T cells, CD11b + cells, and NK cells. (**G**,**H**) B16F10 tumor-bearing mice were treated intravenously two times at a 7-day interval with combination of JCXH-211m (10 μg) and anti-PD1 antibody (3 mg/kg). At 3 days after the second treatment, tumor tissues (n = 8) were harvested and analyzed for (**G**) tumor-infiltrating T cells and (**H**) NK cells by flow cytometry. Individual data points represent mean ± SEM. The difference between vehicle and treatment was analyzed statistically by two-tailed t test. *P < 0.05; **P < 0.01; ***P < 0.001; *ns* not significant (P > 0.05).

**Figure 6:**
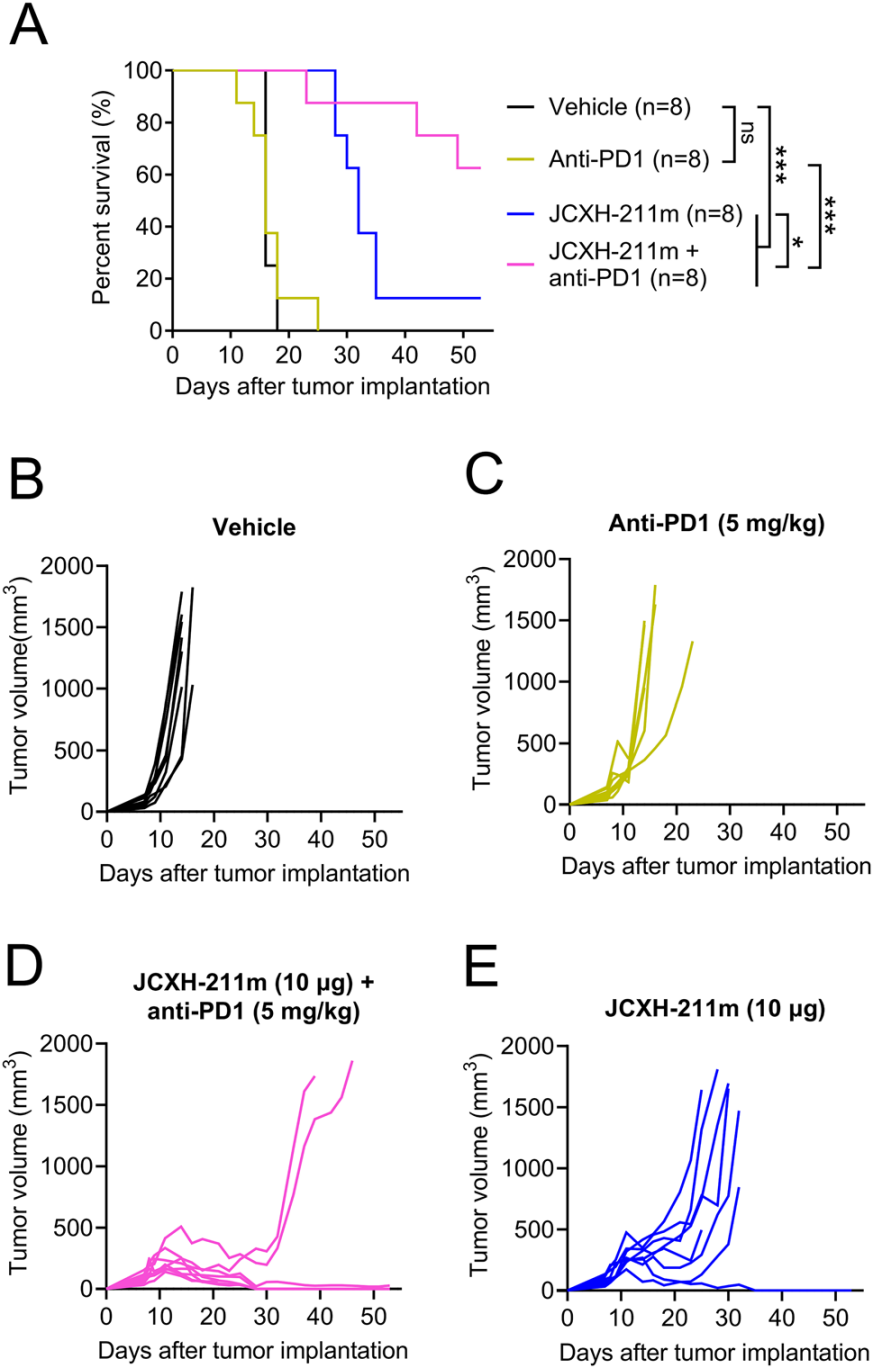
Synergistic antitumor effects of JCXH-211 and anti-PD1 antibody on immune checkpoint inhibitor-resistant B16F10 melanoma. When average tumor volume reached around 85 mm^3^ at day 7 after B16F10 tumor implantation, tumor-bearing mice were treated with anti-PD1 antibody (5 mg/kg, intraperitoneal, eight times, 3–4 days interval), JCXH-211m (10 μg, intravenous, three times, at 14 days interval), or combination of JCXH-211m and anti-PD1 antibody (n = 8, each group). In the vehicle control group, mice were treated with PBS intraperitoneal at 3–4 days interval and PBS intravenous at 14-day interval, corresponding to the anti-PD-1 and JCXH-211m treatments. (**A**) Survival was plotted using the Kaplan–Meier curve and analyzed using the log-rank test. *P < 0.05; ***P < 0.001; *ns* not significant. (**B**–**E**) The tumor volumes of individual mice in the group of indicated treatment regimen were plotted. See also Supplementary Fig. 6.

We next asked whether intravenous treatment with JCXH-211 induced antitumor effects in tumor-bearing mice. It has been demonstrated that B16F10 mouse melanoma had significantly less amounts of tumor-infiltrating T cells than MC38 mouse colon tumor and thus B16F10 mouse melanoma is considered as an immunologically ‘cold’ tumor that is resistant to immune checkpoint inhibitors^22^. Intratumoral treatment with IL-12 srRNA was demonstrated to increase T cells and NK cells in B16F10 tumor and inhibited B16F10 tumor growth^14^. We asked herein whether intravenous treatment with JCXH-211 would induce antitumor effects against B16F10 tumor. We observed that intravenous injection of JCXH-211 significantly inhibited B16F10 melanoma growth in a dose-dependent manner (Fig. 5A). Strikingly, single intravenous administration of JCXH-211 was able to significantly suppress the tumor growth even when the treatment was given to mice with a large tumor around 800 mm^3^ (Fig. 5B). However, no significant increase was recorded in tumor-infiltrating T cells and NK cells in B16F10 tumor-bearing mice with intravenous injections of JCXH-211 (Fig. 5C,D). In addition, ex vivo analysis of splenic T cells in MC38 tumor bearing mice revealed that CD8^+^ IFN-γ producing effector T lymphocytes specific to MC38 tumor cell, but not to B16F10 tumor cells, were significantly augmented after intravenous injection of JCXH-211 but not of saline or blank LNP (Supplementary Fig. 5). Interestingly, intravenous treatment with JCXH-211 significantly decreased the levels of tumor-infiltrating polymorphonuclear myeloid-derived suppressor cells (PMN-MDSCs) in B16F10 tumor-bearing mice (Fig. 5E). Moreover, intravenous treatment with JCXH-211 significantly increased the levels of programmed cell death protein 1 (PD1)-positive T cells and NK cells in the TME of B16F10 tumors (Fig. 5F). PD1 signaling on T cells and NK cells is known to induce apoptosis and reduce survival of activated T cells and NK cells, leading to suppression of antitumor innate and adaptive immune responses^23,24^. Blocking PD1 with anti-PD1 antibody increased the levels of tumor-infiltrating T cells and NK cells in B16F10 tumor-bearing mice with intravenous treatment with JCXH-211 compared to the mice with control treatment (Fig. 5G,H). Furthermore, the combination of JCXH-211 and anti-PD1 antibody significantly improved the survival rate of B16F10 tumor-bearing mice compared to JCXH-211 and anti-PD1 antibody alone (60 vs 12.5 vs 0%, P < 0.05) (Fig. 6). 50% of B16F10 tumor-bearing mice treated with combination of JCXH-211 and anti-PD1 antibody achieved complete response (tumor-free survival) for the duration of the study (53 days) (Fig. 6D).

### Intravenous treatment with JCXH-211 leads to complete response in mice with EMT6 mammary tumors

**Figure 7:**
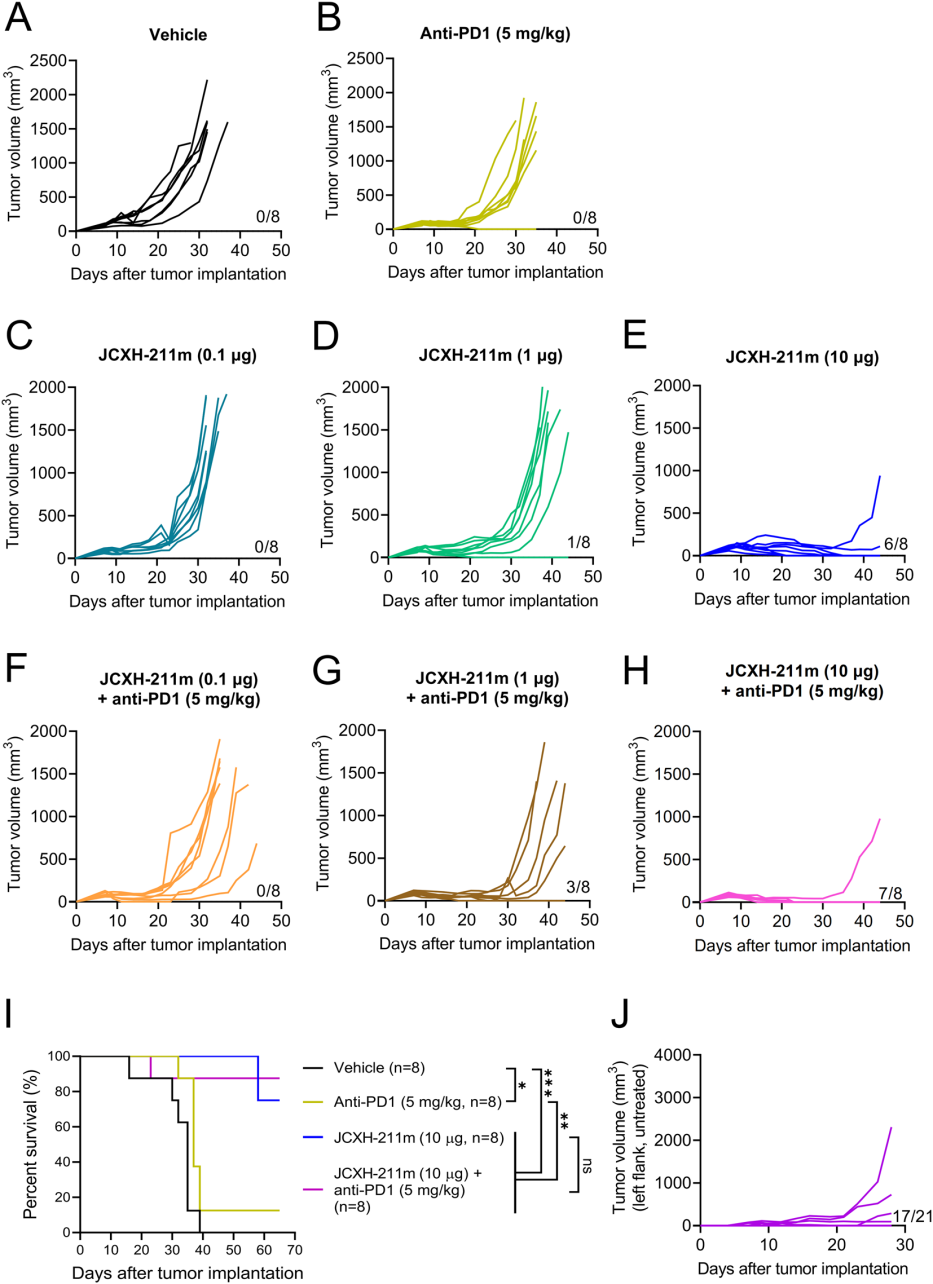
Complete response to JCXH-211 in immune checkpoint inhibitor-sensitive EMT6 breast cancer in an orthotopic tumor model. (**A**–**I**) At day 7 after EMT6 tumor implantation in mammary fat pad, tumor-bearing mice (average tumor volume, 90 mm^3^) were treated PBS control (vehicle), anti-PD1 antibody (5 mg/kg, intraperitoneal, eight times, 3–4 day interval), JCXH-211m (0.1, 1, or 10 μg, intravenous, three times, at 14 day interval), or combination of JCXH-211m and anti-PD1 antibody (n = 8, each group). (**A**–**H**) The tumor volumes of individual mice in the group of indicated treatment regimen were plotted. Number of mice with complete response per total number of mice treated is indicated above the x-axis. (**I**) Survival was plotted using the Kaplan–Meier curve and analyzed using the log-rank test. *P < 0.05; **P < 0.01; ***P < 0.001; *ns* not significant. (**J**) At 44 days after tumor implantation, indicated numbers of mice from JCXH-211m-treated groups in (**A–I**, Supplementary Fig. 6B) showed complete tumor regression and were rechallenged with the same EMT6 tumors on contralateral side. No additional treatment was given after tumor rechallenge. The tumor volumes of individual mice were plotted. See also Supplementary Fig. 6.

As shown in Fig. 6E and Supplementary Fig. 6A, JCXH-211 monotherapy induced complete response in only about 12.5% of mice with the cold B16F10 melanoma although monotherapy significantly delayed the tumor progression as compared to the vehicle control. Next, we asked if JCXH-211 monotherapy would improve the complete response rate in mice with a relatively immunologically ‘hot’ tumors. The EMT6 mammary tumor is known to be inflamed with tumor-infiltrating lymphocytes and sensitive to immune checkpoint inhibitor therapy^25^. Consistent with previous study, anti-PD1 monotherapy significantly inhibited tumor growth in EMT6 tumor-bearing mice compared to control treatment (Fig. 7 and Supplementary Fig. 6B). JCXH-211 monotherapy also inhibited tumor growth in EMT6 tumor-bearing mice in a dose-dependent manner (Fig. 7C–E). Interestingly, EMT6-tumor bearing mice had a sixfold increased complete response rate compared to B16F10 tumor-bearing mice after three treatments with JCXH-211 monotherapy at high dose (Figs. 6D and 7E; P = 0.0019). Surprisingly, single treatment with JCXH-211 induced comparable complete response rate to three treatments with JCXH-211 in EMT6 tumor-bearing mice (Supplementary Fig. 6C). Combination of JCXH-211 and anti-PD1 significantly increased the inhibition of EMT6 tumor growth at low but not high dose of JCXH-211 compared to JCXH-211 monotherapy (Fig. 7C–I). Finally, over 80% of mice with complete response after receiving JCXH-211 alone or in combination with anti-PD1 were resistant to the rechallenge of the same EMT6 tumor cells (Fig. 7J).

## Discussion

In this study, we demonstrated that our novel LNP XH-07-mediated delivery of IL-12 srRNA could effectively and safely induce antitumor responses in syngeneic mouse tumor models and patient-derived colon cancer xenograft models. The antitumor effects of intravenous administration of JCXH-211 were comparable to those of intratumoral administration of JCXH-211. Furthermore, JCXH-211 synergized with anti-PD1 to successfully eradicate “cold” tumors while JCXH-211 monotherapy induced complete regression of “hot” tumors in syngeneic mouse tumor models. In addition, mice with complete response after JCXH-211 therapy were resistant to distal rechallenge of the same tumor cells indicating the induction of systemic immunity against tumor metastasis and/or recurrence.

Because of the enhanced permeability and retention (EPR) effect, nanoparticles penetrate preferentially into tumor tissue through leaky tumor blood vessels, allowing the nanoparticles to release antitumor agents selectively in tumor tissues when administered intravenously^26^. We observed that tumors accumulated significantly higher level of srRNA vector than some normal tissues including kidney, liver, heart, bone marrow, and blood of tumor-bearing mice with intravenous treatment with JCXH-211, as demonstrated by the levels of NSP4. However, spleen, draining lymph node, lung, brain, and ovary of JCXH-211-treated mice accumulated comparable amounts of srRNA vector to tumor within a day after intravenous administration of JCXH-211. A biodistribution study using Cy5-labeled LNP-srRNA revealed that the LNP formulation entered mostly in the liver rather than tumor immediately after intravenous injection. The tumor, spleen, kidney, lung, and heart displayed lower levels of LNP accumulation than liver 2–24 h after injection. These results suggested that the selectively high level of srRNA in tumor tissues was likely a result of preferential srRNA replication rather than LNP retention in the tumor. More interestingly, tumor, but not normal tissues, had much stronger and more durable protein expression of IL-12 srRNA in tumor-bearing mice after intravenous administration of JCXH-211, in vitro transfection experiment supported that tumor cells were more permissive to IL-12 srRNA replication and translation than healthy primary cells.

Specific cell types producing IL-12 in each organ after intravenous administration of JCXH-211 were not assessed in the present study. Such information is also scarce in the literature. The tissue distribution of srRNA-based vaccines was investigated previously by others after intramuscular administration. The data revealed that srRNA was predominantly expressed in the injection site muscle, draining lymph nodes, and spleen^27,28^. It remains elusive whether the srRNA was directly distributed to the lymph nodes and spleen, or it entered the immune cells that subsequently trafficked to the lymph nodes and spleen. The detailed cellular expression profile of srRNA-based therapeutics in these organs warrants further investigation.

Extensive studies have been conducted on the mechanism of action (MOA) of IL-12 in cancer immunotherapy. Firstly, IL-12 can activate T cells and NK cells to drive Th1 differentiation of CD4^+^ T cells via enhanced production of IFN-γ and augments the cytolytic ability of NK and CD8^+^ T cells towards tumor cells via granzyme B and perforin secretion^1^. Through these effects, IL-12 helps to reshape the immunosuppressive TME, and potentiates the recruitment and killing efficiency of cytotoxic cells against tumor cells, mediating tumor regression. Indeed, intratumoral treatment with LNP-srRNA encoding IL-12 increased the infiltration of T cells and NK cells in B16F10 tumor and inhibited B16F10 tumor growth^14^. Choi and his colleagues also found that intratumoral injection of oncolytic adenovirus co-expressing IL-12 and IL-18 in B16F10 tumor bearing mice could enhance the infiltration of T cells and NK cells in the tumor, which conferred potent antitumor effects^29^. In this study, we also observed an increasing trend of tumor-infiltrated T cells and NK cells after JCXH-211 administration, although the data showed significant differences only when combined with anti-PD1 antibodies. Secondly, IL-12 can drive the M1 polarization of macrophages and reprogram the MDSCs towards an antigen-presenting cell phenotype^30^. Moreover, the IL-12-augmented IFN-γ upregulates the expression of major histocompatibility class I and II proteins (MHC-I/MHC-II) on antigen-presenting cells, thereby improving the tumor antigen presentation for recognition by T lymphocytes^31^. In our study, decreased infiltration of PMN-MDSCs in the tumor was observed after intravenous injection of JCXH-211, in consistent with the notion of IL-12 mediated modulation on MDSCs.

Type I IFNs are essential downstream effector molecules of viral RNA- and DNA-sensing PRR signaling for innate immune responses against both viruses and tumors^32,33^. Synthetic srRNA-based cancer therapy can induce type I IFN and IFN-stimulated responses at the site of injection through RNA-sensing PRR signaling^10,34^. In the tumor, type I IFNs are potent immune modulators that can convert immunologically ‘cold’ tumors to ‘hot’ tumors^8,9^, and type I IFN receptor signaling restricts the acquisition of suppressive activity by MDSCs^35^. On the other hand, the type I IFN and RNA-sensing PRR responses may interfere with srRNA expression, and the inhibition of these responses improved srRNA expression in vitro and *in vivo*^36,37^. However, it is still unclear how type I IFNs and RNA-sensing PRR responses interfere with or enhance the therapeutic efficacy of synthetic srRNA. TME downregulates RNA-sensing PRRs and IFN-stimulated genes (ISGs), promoting tumor progression and metastasis ^38–40^. But such a micro-environment probably facilitates the srRNA expression and replication as well. In the current study, we observed that tumor tissues had significantly higher and prolonged expression of IL-12 srRNA compared to normal tissues in tumor-bearing mice with intravenous administration of JCXH-211. We speculated that IL-12 srRNA induced decreased activation of RNA-sensing PRRs and type I IFN responses in tumor tissues compared to in normal tissues in the tumor-bearing mice with JCXH-211 treatment, thereby leading to higher expression and replication of IL-12 srRNA in tumor tissues than in normal tissues. Further studies are warranted to elucidate how tumor and normal tissues differentially express synthetic srRNA.

One potential explanation for poor treatment outcomes of systemic protein-based IL-12 therapy is insufficient delivery of IL-12 at tolerated doses to the TME in cancer patients^41^. Our data suggest that systemic srRNA-LNP complex-based IL-12 therapy can concentrate IL-12 in tumor tissues by multiple mechanisms, including the innate immune-suppressive TME, and self-replication property of srRNA, which make tumors exposed to a high and effective dose of IL-12. Intratumorally administered JCXH-211 has already entered phase I clinical trials for patients with cutaneous cancers or advanced solid cancers (NCT05539157 and NCT05727839). Based on the current findings, we expect that intravenously administered JCXH-211 will be a potential option for treatment of cancer patients who are not eligible for intratumoral IL-12 therapy. It will be advantageous because intravenous treatment can be applied without the need for special medical equipment such as imaging guiding systems and the staff to operate them, thus accessible in those community-based medical facilities.

## Methods

### Ethics statement

The PDX mouse tumor model study was performed in WuXi AppTec (Shanghai, China); the in vivo efficacy and pharmacokinetic (PK)/pharmacodynamic (PD) studies in tumor-bearing mice were performed at BioDuro-Sundia (Shanghai, China). All the experimental procedures were in accordance with local regulations and guidelines and were approved by an Institutional Animal Care and Use Committee (IACUC) in WuXi AppTec and BioDuro-Sundia (Shanghai), respectively. The tolerability study in reused cynomolgus monkeys was performed at JOINN LABORATORIES (Suzhou) Inc. The animal care and the experimental procedures were compliant with the SOPs of JOINN LABORATORIES (Suzhou) Inc. and approved by the IACUC at JOINN LABORATORIES (Suzhou) Inc.

All experiments in this study were in accordance with ARRIVE guidelines 2.0. All the pictures used in figures comply with the digital image and integrity policies.

### Cell lines and animals

MC38, B16F10 and EMT6 cell lines were obtained from ATCC (Manassas, VA, USA) and maintained according to ATCC's instructions. All cell lines were routinely checked for mycoplasma contamination. C57BL/6 and BALB/c mice were obtained from Shanghai Lingchang Biological Technology (Shanghai, China) and used for syngeneic mouse tumor models. NOG (NOD/Shi-scid, IL-2Rγnull) mice were obtained from Wuxi AppTec (Shanghai, China) and used for patient-derived xenograft models. All in vivo experiments were performed in accordance with the Institutional Animal Care and Use Committee at BioDuro-Sundia (Shanghai, China) and WuXi AppTec. Tumor-bearing mice were euthanized by CO_2_ inhalation and cervical dislocation.

### JCXH-211 production

Synthesis of IL-12 srRNA and formulation of IL-12 srRNA-LNP were performed using methods as described previously^42^. Briefly, linear DNA templates were generated by digesting plasmid DNA with BspQI restriction enzyme (NEB, MA, USA), then purified by phenol and chloroform. IL-12 srRNA was generated by in vitro transcription (IVT) using linear plasmid DNA template (50 μg/ml) and IVT reaction mixture containing IVT Reaction Buffer, MgCl_2_ (6 mM), ribonucleoside triphosphate mix (6 mM each), yeast inorganic pyrophosphatase (2 U/ml), RNase inhibitor (1,000 U/ml), and T7 RNA Polymerase (5,000 U/ml) (all from Hongene Biotech, Shanghai, China). DNA templates in the transcribed srRNA were removed by Turbo DNase (Invitrogen, MA, USA). The transcribed IL-12 srRNA was capped using the following components: vaccinia capping system (500 U/ml), capping buffer, GTP (0.5 mM), S-adenosylmethionine (0.128 mM), RNase inhibitor (667 U/ml), and 2´-O-methyltransferase (2500 U/ml) (all from Hongene Biotech, Shanghai, China). The capping reaction was carried out by incubating srRNA with Vaccinia virus capping enzyme (Hongene Biotech, Shanghai, China) at 37 ± 2 °C for 90 min, which added a 7-methylguanylate cap structure (Cap 0) to the 5’ end of srRNA. RNA was purified by LiCl (Invitrogen, MA, USA) precipitation, followed by analysis using agarose gel electrophoresis and liquid chromatography-mass spectrometry. To formulate IL-12 srRNA-LNP complex, LNPs were formulated by rapid mixing of ethanol phase and aqueous phase using a microfluidic device (INano™ L system, Micro & Nano). The aqueous phase contained citrate buffer (pH 6.0, 50 mM) and IL-12 srRNA. The ethanol phase comprised XH-07 (Immorna Biotechnology, Hangzhou, China), cholesterol (Jiangsu Southeast Nanomaterials, Jiangsu, China), 1,2-diastearoyl-sn-glycero-3-phosphocholine (Jiangsu Southeast Nanomaterials), and 1,2-dimyristoyl-rac-glycero-3-methoxypolyethyleneglycol-2000 (SINOPEG, Fujian, China). The srRNA-LNPs were assembled with these four lipid components mixed at a molar ratio of 40:48:10:2.0 and a lipid/RNA (N/P) ratio of 6. The generated IL-12 srRNA-LNP was then concentrated by tangential flow filtration (TFF) and buffer-exchanged to the final formulation (20 mmol/L Tris, 5 mmol/L NaCl, 7.5% sucrose, pH 7.2), and characterized with respect to particle size, polydispersity index, and srRNA. The average diameter of these srRNA-LNPs was approximately 80 nm with a polydispersity index of 0.07–0.16 and an encapsulation efficiency of > 85%. The ionizable cationic lipid XH-07 and the srRNA sequence used in JCXH-211 are described in the international patent application “INTERLEUKIN-12 SELF-REPLICATING RNA AND METHODS”, PCT/CN2022/139738. The structure of XH-07 and the sequence of srRNA mouse IL-12 plus srRNA human IL-12 used in this study was shown in Supplementary Table 1.

### Biodistribution of JCXH-211 in mouse organs after i.v. administration

1 × 10^6^ MC38 cells in 100 μl phosphate-buffered saline (PBS) was implanted subcutaneously in the right flank of female C57BL/6 mice. 6 days later, tumor-bearing mice were intravenously injected with srRNA encapsulated with Cy5-labeled LNP at a dose of 0.2 mg/kg srRNA. Ex vivo imaging (Cy5.5 channel) was conducted at 2-, 4-, and 24-h post injection using the IVIS^®^ Lumina III In Vivo Imaging System (PerkinElmer).

### In vitro transfection with IL-12 srRNA

Mouse primary cells (colon epithelial cells and splenocytes) were isolated from colon tissues and spleen tissues of C57BL/6 mice. Colon epithelial cells were cultured in DMEM medium supplemented with 10% FBS plus 1% Penicillin–Streptomycin and were expanded for 14 days after removing the mixed fibroblasts by one round of differential adhesion and two rounds of differential trypsinization. Splenocytes were re-suspended in RPMI1640 medium (Cytiva, Marlborough, MA, USA) supplemented with 10% FBS plus 1% Penicillin–Streptomycin, then they were subjected to transfection experiment without further expansion.

One day before transfection, 8 × 10^4^ adherent cells (B16F10, MC38, EMT6 or colon epithelial cells) were seeded in one well of 24 well plates in 0.5 mL DMEM medium supplemented with 10% FBS plus 1% Penicillin–Streptomycin. 2 × 10^6^ fresh splenocytes in 0.5 mL RPMI-1640 medium supplemented with 10% FBS plus 1% Penicillin–Streptomycin were loaded in 24 well plates before the transfection. Each type of cells was transfected with 50 ng or 500 ng srRNA encoding IL-12p70 using Lipofectamine MessengerMax (Invitrogen, USA) or with 50 ng or 500 ng JCXH-211m after LNP encapsulation (Immorna Biotechnology, Hangzhou, China). Supernatants were collected at 48 h post the transfection. The level of mouse IL-12p70 in supernatants was determined using a commercial ELISA kit (Cat No: EMC006, Neobioscience, Shenzhen, Guangdong Province, China) following the manufacturer’s instructions.

### Syngeneic mouse tumor models and antitumor therapy

All animals were specific pathogen free (SPF). Mouse tumor cells in exponential growth phase were harvested and implanted into syngeneic mice. 3 × 10^5^ MC38 or B16F10 cells in 100 μl phosphate-buffered saline (PBS) were implanted subcutaneously in the right flank of female C57BL/6 mice. For the mammary carcinoma model, 5 × 10^5^ EMT6 cells in 100 μl PBS were implanted in the left mammary fat pad of female BALB/c mice. For tumor rechallenge studies, 3 × 10^5^ MC38 were implanted in the opposite flank of primary MC38 tumor and 5 × 10^5^ EMT6 cells were implanted in the opposite mammary fat pad of primary EMT6 tumor after complete eradication of primary tumor. The tumor volume was measured by calipers and calculated by the formula: 0.5 × (height) × (width^2^). When primary tumor volume reached 80–120 mm^3^, tumor-bearing mice were assigned into groups using an Excel-based randomization software performing stratified randomization based on their tumor volumes, then treated with JCXH-211 alone or combination with anti-PD1 antibody. JCXH-211 at various doses was administered either intratumorally or intravenously. Anti-PD1 antibody (3 or 5 mg/kg; BioXcell, Lebanon, NH) was administered intraperitoneally. Survival was defined as the time from first treatment to death or the date of euthanization due to tumor burden (≥ 3000 mm^3^) by CO_2_ inhalation and cervical dislocation.

### PDX tumor model

30 mm^3^ tumor pieces from a single human colon cancer patient (WuXi AppTec) were implanted subcutaneously in the right flank of NOG mice. When the average tumor volume reached around 90 mm^3^ on day 24 after tumor implantation, tumor-bearing mice received 3 × 10^6^ human peripheral blood mononuclear cells (HemaCare, Los Angeles, CA) peritumorally for humanization of the mice, followed by intratumoral treatment with JCXH-211 at various doses. The donor PBMC was selected based on successful engraftment in NOG mice. However, there was no MHC matching between the patient-derived tumor and the PBMC in this PDX tumor model. In this study, tumor-bearing mice were euthanized by CO_2_ inhalation and cervical dislocation.

### Analysis of tumor-infiltrated immune cells

Single cell suspension was obtained from tumor tissues using the gentleMACS™ C Tubes (Miltenyi Biotec, Shanghai, China) in combination with the gentleMACS™ Dissociator (Miltenyi Biotec) as following manufacturer's instructions. Cells were incubated with either FcR blocking reagent (Miltenyi Biotec) or mouse BD Fc Block™ (BD Biosciences, San Jose, CA) to block non-specific binding of antibodies, followed by cell staining with antibodies listed in Supplementary Table 5. For cell surface staining, cells were stained in PBS containing 2% fetal bovine serum. For intracellular staining, cells were stained using the Transcription Factor Staining Buffer Set (eBioscience, San Diego, CA). After staining, cells were analyzed using BD Fortessa flow cytometer (BD Biosciences). FlowJo 10 software was used for data analysis with gating strategies shown in Supplementary Fig. 7.

### Pharmacokinetics of JCXH-211 in tumor-bearing mice

B16F10 tumor-bearing mice were sacrificed by CO_2_ inhalation and cervical dislocation at various time points after treatment with either JCXH-211 or vehicle control. Tumor, plasma, and organs were harvested, wet-weighed, and analyzed for biodistribution of IL-12 srRNA. To this end, we performed reverse transcription-quantitative polymerase chain reaction (RT-qPCR) to determine relative expression of NSP4. For RT reaction, total RNA was isolated from plasma and tissues using QIAamp RNA blood mini kit and RNeasy mini kit (both from Qiagen China, Shanghai, China), respectively, followed by RT using random primers and MultiScribe™ reverse transcriptase (both from Thermo Fisher Scientific, Waltham, MA). The relative expression level of NSP gene was measured by real-time PCR using cDNA, TaqMan™ Gene Expression Master Mix (Thermo Fisher Scientific), Power SYBR Green Master Mix (Thermo Fisher Scientific), NSP4 forward primer (5’-GTCCAGGAAGGTGGAGAACA-3’), and NSP4 reverse primer (5’-AAGGCACGGTTCACACTAGA-3’). The level of glyceraldehyde-3-phosphate dehydrogenase (GAPDH) gene was measured as a reference gene using GAPDH forward primer (5’-AGGTCGGTGTGAACGGATTTG-3’) and GAPDH reverse primer (5’-TGTAGACCATGTAGTTGAGGTCA-3’).

### Tolerability of JCXH-211 in cynomolgus monkeys after intravenous administration

Two cynomolgus monkeys (one male and one female, 2.4–2.5 years old, reused) were intravenously administered with JCXH-211 at 100 μg once a week for 3 consecutive weeks. The day with the first dosing was defined as day (D) 1, the other two doses were given on D 8 and D 15. Clinical observation, injection site reactions, body weights, body temperature, food consumption, hematology, coagulation, electrocardiogram, and blood chemistry were monitored according to a predefined protocol at JOINN LABORATORIES (Suzhou) Inc.

### Analysis of IL-12, IFN-γ, and AST in tumor tissues and serum

Assessment of cytokines was conducted in individual animals. The baseline was determined in control tumor-bearing mice at the beginning of treatment (PBS). 50 mg of tumor tissues were lysed with RIPA Buffer (MilliporeSigma, Burlington, MA) containing protease inhibitor cocktail (MilliporeSigma) and phosphatase inhibitor cocktail 2 (MilliporeSigma) to collect proteins for the enzyme-linked immunosorbent assay (ELISA). Total protein amounts were determined using BCA protein assay kit (Thermo Fisher Scientific). The levels of IL-12 and IFN-γ in tumor tissues and serum were determined using mouse IL-12 p70 Quantikine ELISA kit (Bio-Techne, Minneapolis, MN) and mouse IFN-γ Quantikine ELISA kit (Bio-Techne), respectively, according to manufacturer’s instruction. The cytokine content in tissues was normalized as picograms of cytokine per gram of tissue. The level of aspartate aminotransferase (AST) in serum was determined using mouse AST assay kit (Abcam China, Shanghai, China).

### Ex vivo analysis of tumor-specific T cells

Female C57BL/6 mice bearing a subcutaneous MC38 tumor of 150–200 mm^3^ were injected once intravenously with vehicle (saline), blank LNP or JCXH-211m (10 μg). Spleens were collected 2 weeks after treatment. Single splenocyte suspension (2 × 10^6^) was co-cultured with mitomycin C-treated (MedChemExpress, NJ, USA) MC-38 or B16F10 tumor cells (2 × 10^4^) in presence of anti-CD28 and anti-CD49d antibodies (BD Pharmingen, NJ, USA) for 18 h. Golgi transporter inhibitor (Invitrogen) was added in the co-culture during the last 16 h of incubation. The percentage of CD8 + IFN-γ + and CD4 + IFN-γ + cells were determined by flow cytometry after performing intracellular cytokine staining. Data are presented as mean ± SD, each dot represents one individual animal (n = 6 per group).

### Statistical analysis

Statistical significance between and among groups was determined by one-way or two-way ANOVA multiple-comparison test or two-tailed t test. Survival significance between groups was determined by Log-rank (Mantel-Cox) test. All the statistical tests were performed using GraphPad Prism (version 9.5.0, GraphPad Software, Boston, MA).

### Supplementary Information

Supplementary Information.

## Data Availability

The data related to this study are available from the corresponding author upon reasonable request, with the permission of Immorna Biotechnology/Immorna Biotherapeutics.
