# Supplementary material for: Intravenous administration of IL-12 encoding self-replicating RNA-lipid nanoparticle complex leads to safe and effective antitumor responses
Source: Sci Rep. 2024 Mar 28;14:7366. doi: 10.1038/s41598-024-57997-w (PMC10978917; doi:10.1038/s41598-024-57997-w)
Supplement: Supplementary file 1 — Supplementary Information. [file 41598_2024_57997_MOESM1_ESM.pdf]

Supplementary Material

Intravenous administration of IL-12 encoding self-replicating RNA-lipid nanoparticle complex leads to safe and effective antitumor responses

Zihao Wang[1*], Yanni Chen[2], Hongyue Wu[1], Min Wang[1], Li Mao[2], Xingdong Guo[2], Jianbo Zhu[1], Zilan Ye[1], Xiaoyan Luo[1], Xiurong Yang[1], Xueke Liu[1], Junhao Yang[1], Zhaolang Sheng[2], Jaewoo Lee[3], Zhijun Guo[1], Yuanqing Liu[2]

[1]Immorna (Hangzhou) Biotechnology, Co. Ltd.; Hangzhou 311215, Zhejiang, China

[2]Immorna (Shanghai) Biotechnology, Co. Ltd.; Shanghai 201199, China

[3]Immorna Biotherapeutics, Inc.; Morrisville 27560, NC, USA

*Corresponding author. Email: Zihao.Wang@immornabio.com;

1 Supplementary Figures and Tables

1.1 Supplementary Figures

A

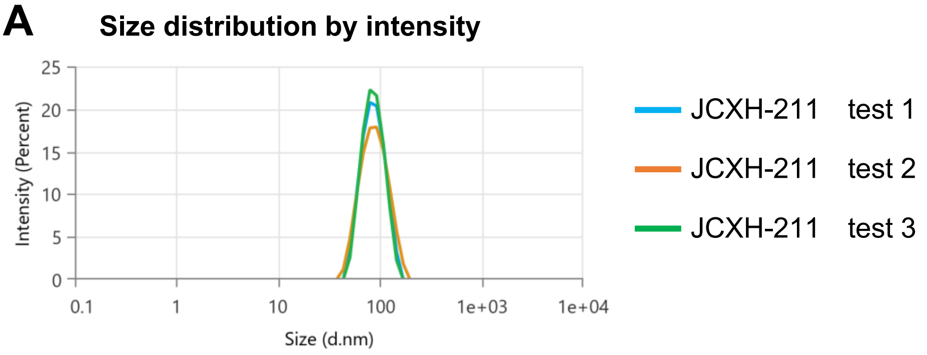

Sample	Size (nm)	Mean size (nm)	Standard Deviation	RSD
JCXH-211 test 1	81.70	82.12	0.5154	0.6277
JCXH-211 test 2	82.69			
JCXH-211 test 3	81.95			

B

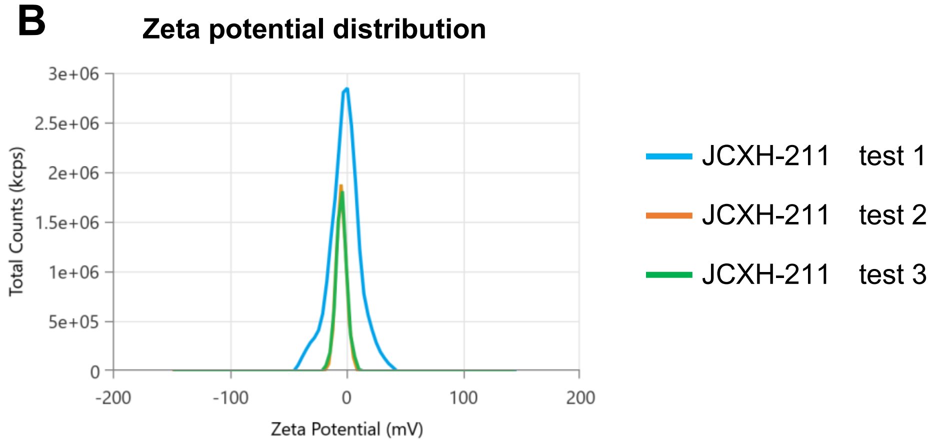

Sample	Zeta potential (mV)	Mean Zeta potential (mV)	Standard Deviation	RSD
JCXH-211 test 1	-1.266	-3.181	1.66	52.17
JCXH-211 test 2	-4.196			
JCXH-211 test 3	-4.081			

Supplementary Figure 1. The characteristics of JCXH-211. (A) The particle size of JCXH-211. (B) The Zeta potential of JCXH-211.

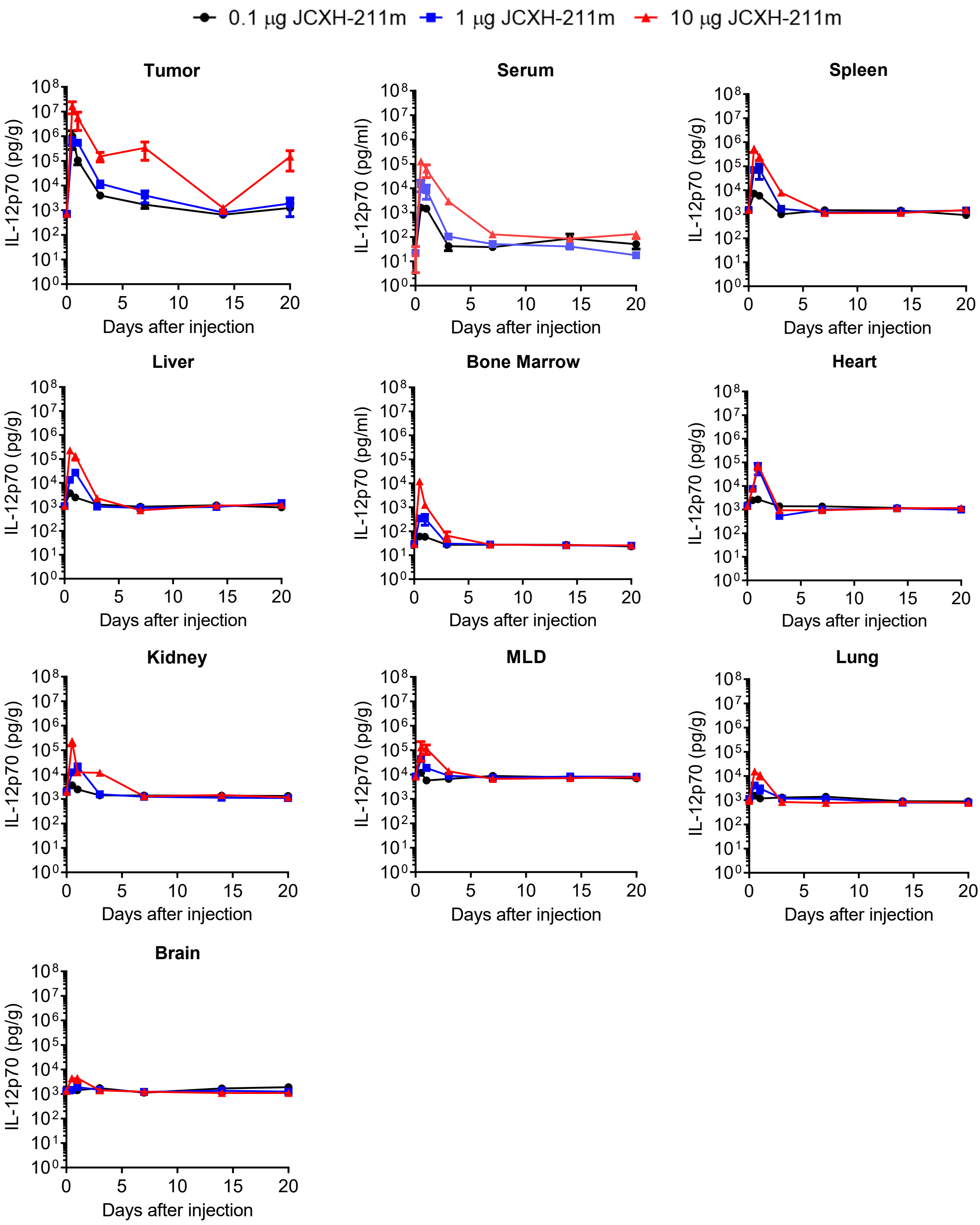

Supplementary Figure 2. IL-12p70 production induced by intravenous administration of JCXH-211. B16F10 tumor-bearing mice were treated once intravenously with JCXH-211m (10 µg). Tumors, sera, and organs were isolated right before and at 12, 24, 72, 168, 336 and 504 h after JCXH-211m treatment. The amounts of IL-12p70 proteins per 1 g tissue weight or 1 mL serum or 1 mL bone marrow were plotted. The cytokine levels before and at indicated time points after JCXH-211m treatment are shown.

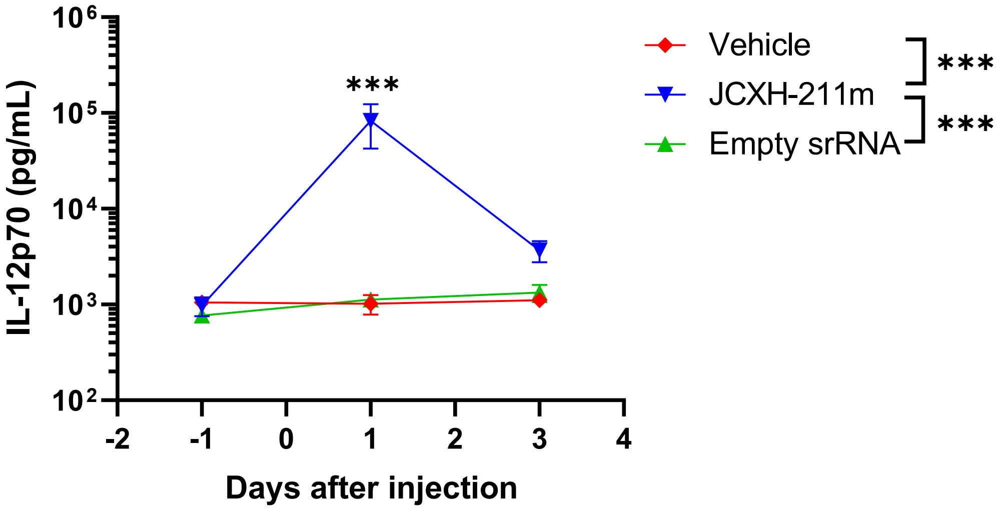

Supplementary Figure 3. IL-12p70 production after intravenous administration of empty srRNA vector-LNP complex or JCXH-211. B16F10 tumor-bearing mice were treated once intravenously with vehicle (PBS), empty srRNA vector-LNP complex or JCXH-211m (10 µg). Sera were collected at D-1, D1 and D3 after treatment. The amounts of IL-12p70 proteins per 1 mL serum were plotted. The cytokine levels before and at indicated time points after indicated treatment were plotted and analyzed using two-way ANOVA with multiple comparisons test.

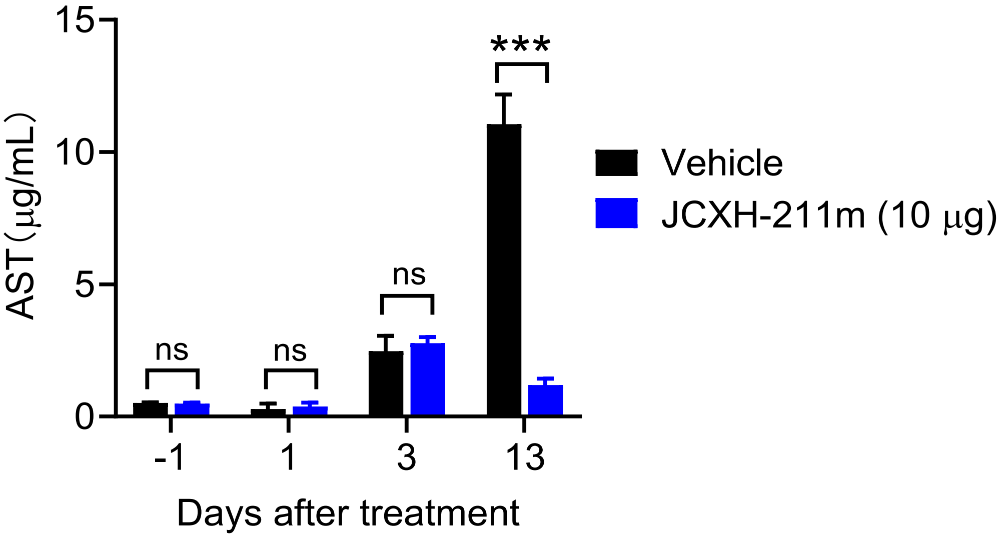

Supplementary Figure 4. Liver toxicity induced by intravenous administration of JCXH-211. B16F10 tumor-bearing mice were treated once intravenously with JCXH-211m (10 μg). Sera were isolated at 1 day before and at 1, 3, and 13 days after JCXH-211m treatment or control treatment (vehicle). The concentrations of serum AST were compared between vehicle-treated and JCXH-211m-treated mice by two-tailed *t* test at indicated time points (n = 8). *** $P < 0.001$.

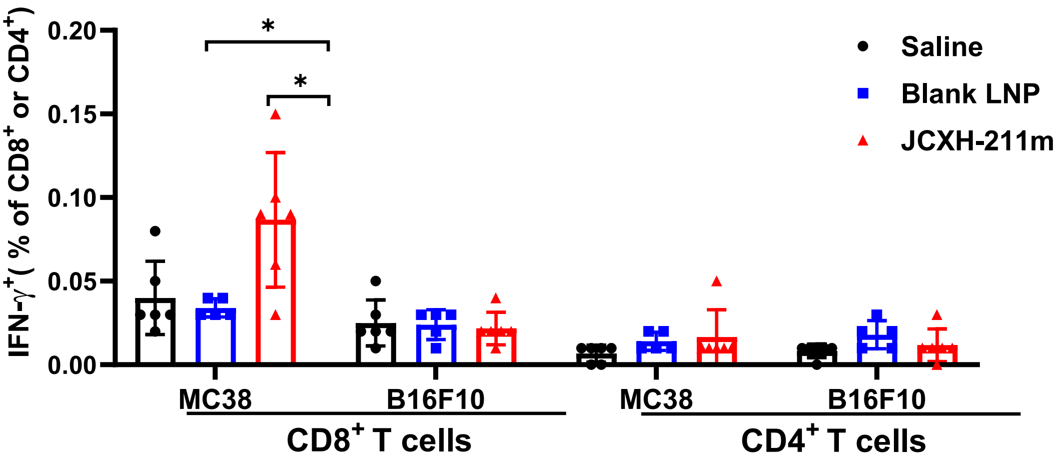

Supplementary Figure 5. *Ex vivo* analysis of tumor-specific T cells in MC38 tumor bearing mice. MC38 tumor-bearing mice were injected once intravenously with vehicle (saline), blank LNP or JCXH-211m (10 μg). Spleens were collected 2 weeks after treatment. Splenocytes (2×10^6) were co-cultured with mitomycin C-treated MC-38 or B16F10 tumor cells (2×10^4) in presence of anti-CD28 and anti-CD49d antibodies for 18 hours. Golgi transporter inhibitor was added in the co-culture during the last 16 hours of incubation. The percentages of CD8+ IFN-γ+ and CD4+ IFN-γ+ cells were determined by flow cytometry after performing intracellular cytokine staining. Data are presented as mean ± SD, each dot represents one individual animal (n = 6 per group). Pairwise differences were statistically analyzed by Multiple *t* tests.

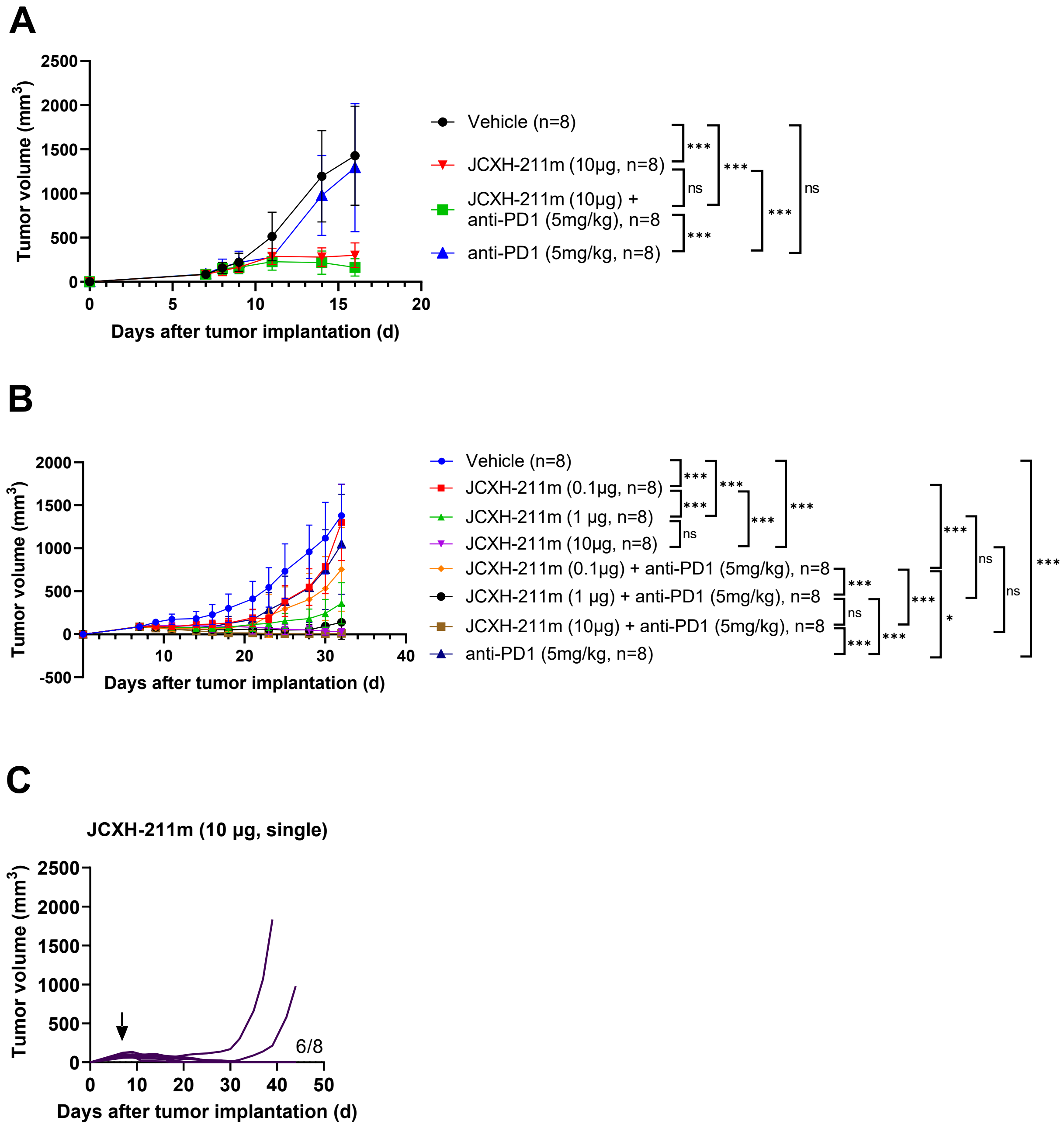

Supplementary Figure 6. Antitumor effects of JCXH-211 and anti-PD1 antibody in murine B16F10 melanoma and EMT6 breast cancer models. (**A**) B16F10 cells were implanted subcutaneously in female C57BL/6 mice. When average tumor volume reached around 85 mm^3 at day 7 after tumor implantation, tumor-bearing mice were treated with PBS control (vehicle), anti-PD1 antibody (5 mg/kg, intraperitoneal, eight times, 3-4 days interval), JCXH-211m (10 µg, intravenous, three times, at 14 days interval), or combination of JCXH-211m and anti-PD1 antibody (n = 8 per group). (**B**) EMT6 cells were implanted in the mammary fat pad of female BALB/c mice. At day 7 after tumor implantation, tumor-bearing mice (average tumor volume, 90 mm^3) were treated with PBS control (vehicle), anti-PD1 antibody (5 mg/kg, intraperitoneal, eight

times, 3-4 days interval), JCXH-211m (0.1, 1, or 10 μg, intravenous, three times, at 14 days interval), or combination of JCXH-211m and anti-PD1 antibody (n = 8 per group). Aggregated tumor growth of each group was plotted and analyzed using two-way ANOVA with multiple comparison test. * $P < 0.05$; ** $P < 0.01$; *** $P < 0.001$; ns: not significant. (**C**) The tumor volumes of individual mice in the group with single injection of JCXH-211m in EMT6 tumor-bearing mice were plotted. Number of mice with complete response per total number of mice treated is indicated above the x-axis.

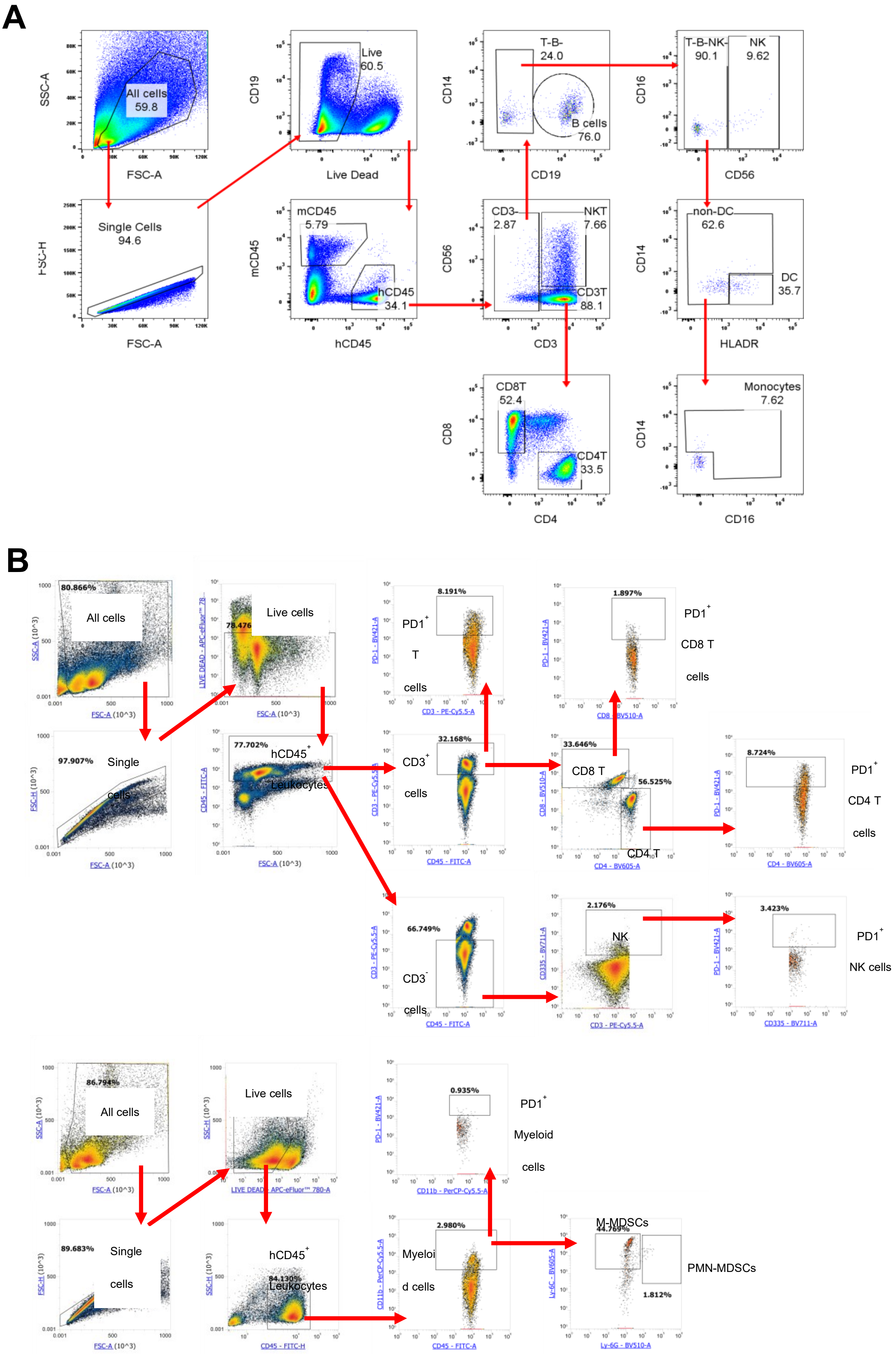

A
All cells 59.8
SSC-A
FSC-A
Single Cells 94.6
FSC-H
Live 60.5
CD19
Live Dead
mCD45 5.79
hCD45 34.1
mCD45
hCD45
T-B- 24.0
B cells 76.0
CD14
CD19
CD3- 2.87
NKT 7.66
CD3T 88.1
CD56
CD3
CD8T 52.4
CD4T 33.5
CD8
CD4
T-B-NK- 90.1
NK 9.62
CD16
CD56
non-DC 62.6
DC 35.7
CD14
HLADR
Monocytes 7.62
CD14
CD16
B
All cells
80.866%
Live cells
78.476
Single cells
97.907%
hCD45+ Leukocytes
77.702%
PD1+ T cells
8.191%
CD3+ cells
32.168%
CD3- cells
66.749%
PD1+ CD8 T cells
1.897%
CD8 T
33.646%
CD4 T
56.525%
PD1+ CD4 T cells
8.724%
NK
2.176%
PD1+ NK cells
3.423%
All cells
86.794%
Live cells
Single cells
89.683%
hCD45+ Leukocytes
84.130%
PD1+ Myeloid cells
0.935%
Myeloid cells
2.980%
M-MDSCs
44.769%
PMN-MDSCs
1.812%

Supplementary Figure 7. Gating strategies in flow cytometry analysis of tumor-infiltrating immune cells. (**A**) Gating strategy for analysis of tumor-infiltrating human lymphocytes in xenograft tumors. (**B**) Gating strategy for analysis of tumor-infiltrating immune cells in B16F10 tumors.

1.2 Supplementary Tables

Supplementary Table 1. The structure of ionizable cationic lipid XH-07 and the sequence of srRNA mouse IL-12 plus srRNA human IL-12 used in this study.

The structure of ionizable cationic lipid XH-07 (Lipid #4 in patent)	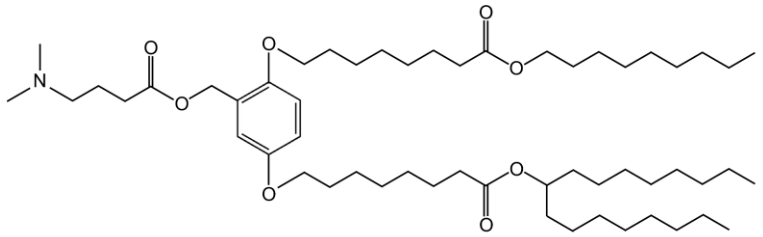<br>**Lipid #4**
srRNA mouse IL-12 sequence<br><br>(Mouse IL-12 sequence is in uppercase letters)	ataggcggcgcatgagagaagcccagaccaattacctacccaaaatggagaaagttcacgttgacatcga<br>ggaagacagcccattcctcagagctttgcagcggagcttcccgcagtttgaggtagaagccaagcaggtc<br>actgataatgaccatgctaatgccagagcgttttcgcatctggcttcaaaactgatcgaaacggaggtgg<br>acccatccgacacgatccttgacattggaagtgcgcccgcccgcagaatgtattctaagcacaagtatca<br>ttgtatctgtccgatgagatgtgcggaagatccggacagattgtataagtatgcaactaagctgaagaaa<br>aactgtaaggaaataactgataaggaattggacaagaaaatgaaggagctcgccgccgtcatgagcgacc<br>ctgacctggaaactgagactatgtgcctccacgacgacgagtcgtgtcgctacgaagggcaagtcgctgt<br>ttaccaggatgtatacgcggttgacggaccgacaagtctctatcaccaagccaataagggagttagagtc<br>gcctactggataggctttgacaccaccccttttatgtttaagaacttggctggagcatatccatcatact<br>ctaccaactgggccgacgaaaccgtgttaacggctcgtaacataggcctatgcagctctgacgttatgga<br>gcggtcacgtagagggatgtccattcttagaaagaagtatttgaaaccatccaacaatgttctattctct<br>gttggctcgaccatctaccacgagaagagggacttactgaggagctggcacctgccgtctgtatttcact<br>tacgtggcaagcaaaattacacatgtcggtgtgagactatagttagttgcgacgggtacgtcgttaaaag<br>aatagctatcagtccaggcctgtatgggaagccttcaggctatgctgctacgatgcaccgcgagggattc<br>ttgtgctgcaaagtgacagacacattgaacggggagagggtctcttttcccgtgtgcacgtatgtgccag<br>ctacattgtgtgaccaaatgactggcatactggcaacagatgtcagtgcggacgacgcgcaaaaactgct<br>ggttgggctcaaccagcgtatagtcgtcaacggtcgcacccagagaaacaccaataccatgaaaaattac<br>cttttgcccgtagtggcccaggcatttgctaggtgggcaaaggaatataaggaagatcaagaagatgaaa<br>ggccactaggactacgagatagacagttagtcatggggtgttgttgggcttttagaaggcacaagataac<br>atctatttataagcgcccggatacccaaaccatcatcaaagtgaacagcgatttccactcattcgtgctg<br>cccaggataggcagtaacacattggagatcgggctgagaacaagaatcaggaaaatgttagaggagcaca<br>aggagccgtcacctctcattaccgccgaggacgtacaagaagctaagtgcgcagccgatgaggctaagga<br>ggtgcgtgaagccgaggagttgcgcgcagctctaccacctttggcagctgatgttgaggagcccactctg<br>gaagccgatgtcgacttgatgttacaagaggctggggccggctcagtggagacacctcgtggcttgataa<br>aggttaccagctacgatggcgaggacaagatcggctcttacgctgtgctttctccgcaggctgtactcaa<br>gagtgaaaaattatcttgcatccaccctctcgctgaacaagtcatagtgataacacactctggccgaaaa<br>gggcgttatgccgtggaaccataccatggtaaagtagtggtgccagagggacatgcaatacccgtccagg<br>actttcaagctctgagtgaaagtgccaccattgtgtacaacgaacgtgagttcgtaaacaggtacctgca<br>ccatattgccacacatggaggagcgctgaacactgatgaagaatattacaaaactgtcaagcccagcgag<br>cacgacggcgaatacctgtacgacatcgacaggaaacagtgcgtcaagaaagaactagtcactgggctag<br>ggctcacaggcgagctggtggatcctcccttccatgaattcgcctacgagagtctgagaacacgaccagc<br>cgctccttaccaagtaccaaccataggggtgtatggcgtgccaggatcaggcaagtctggcatcattaaa<br>agcgcagtcaccaaaaaagatctagtggtgagcgccaagaaagaaaactgtgcagaaattataaggacg<br>tcaagaaaatgaaagggctggacgtcaatgccagaactgtggactcagtgctcttgaatggatgcaaaca<br>ccccgtagagaccctgtatattgacgaagcttttgcttgtcatgcaggtactctcagagcgctcatagcc<br>attataagacctaaaaaggcagtgctctgcggggatcccaaacagtgcggtttttttaacatgatgtgcc<br>tgaaagtgcattttaaccacgagatttgcacacaagtcttccacaaaagcatctctcgccgttgcactaa<br>atctgtgacttcggtcgtctcaaccttgttttacgacaaaaaaatgagaacgacgaatccgaaagagact<br>aagattgtgattgacactaccggcagtaccaaacctaagcaggacgatctcattctcacttgtttcagag<br>ggtgggtgaagcagttgcaaatagattacaaaggcaacgaaataatgacggcagctgcctctcaagggct<br>gacccgtaaaggtgtgtatgccgttcggtacaaggtgaatgaaaatcctctgtacgcacccacctcagaa<br>catgtgaacgtcctactgacccgcacggaggaccgcatcgtgtggaaaacactagccggcgacccatgga<br>taaaaacactgactgccaagtaccctgggaatttcactgccacgatagaggagtggcaagcagagcatga<br>tgccatcatgaggcacatcttggagagaccggaccctaccgacgtcttccagaataaggcaaacgtgtgt<br>tgggccaaggctttagtgccggtgctgaagaccgctggcatagacatgaccactgaacaatggaacactg<br>tggattattttgaaacggacaaagctcactcagcagagatagtattgaaccaactatgcgtgaggttctt<br>tggactcgatctggactccggtctattttctgcacccactgttccgttatccattaggaataatcactgg<br>gataactccccgtcgcctaacatgtacgggctgaataaagaagtggtccgtcagctctctcgcaggtacc<br>cacaactgcctcgggcagttgccactggaagagtctatgacatgaacactggtacactgcgcaattatga<br>tccgcgcataaacctagtacctgtaaacagaagactgcctcatgctttagtcctccaccataatgaacac

	ccacagagtgacttttcttcattcgtcagcaaattgaagggcagaactgtcctggtggtcggggaaaagt<br>tgtccgtcccaggcaaaatggttgactggttgtcagaccggcctgaggctaccttcagagctcggctgga<br>tttaggcatcccaggtgatgtgcccaaatatgacataatatttgttaatgtgaggaccccatataaatac<br>catcactatcagcagtgtgaagaccatgccattaagcttagcatgttgaccaagaaagcttgtctgcatc<br>tgaatcccggcggaacctgtgtcagcataggttatggttacgctgacagggccagcgaaagcatcattgg<br>tgctatagcgcggcagttcaagttttcccgggtatgcaaaccgaaatcctcacttgaagagacggaagtt<br>ctgtttgtattcattgggtacgatcgcaaggcccgtacgcacaatccttacaagctttcatcaaccttga<br>ccaacatttatacaggttccagactccacgaagccggatgtgcaccctcatatcatgtggtgcgaggga<br>tattgccacggccaccgaaggagtgattataaatgctgctaacagcaaaggacaacctggcggaggggtg<br>tgcggagcgctgtataagaaattcccggaaagcttcgatttacagccgatcgaagtaggaaaagcgcgac<br>tggtcaaaggtgcagctaaacatatcattcatgccgtaggaccaaacttcaacaaagtttcggaggttga<br>aggtgacaaacagttggcagaggcttatgagtccatcgctaagattgtcaacgataacaattacaagtca<br>gtagcgattccactgttgtccaccggcatcttttccgggaacaaagatcgactaacccaatcattgaacc<br>atttgctgacagctttagacaccactgatgcagatgtagccatatactgcagggacaagaaatgggaaat<br>gactctcaaggaagcagtggctaggagagaagcagtggaggagatatgcatatccgacgactcttcagtg<br>acagaacctgatgcagagctggtgagggtgcatccgaagagttctttggctggaaggaagggctacagca<br>caagcgatggcaaaactttctcatatttggaagggaccaagtttcaccaggcggccaaggatatagcaga<br>aattaatgccatgtggcccgttgcaacggaggccaatgagcaggtatgcatgtatatcctcggagaaagc<br>atgagcagtattaggtcgaaatgccccgtcgaagagtcggaagcctccacaccacctagcacgctgcctt<br>gcttgtgcatccatgccatgactccagaaagagtacagcgcctaaaagcctcacgtccagaacaaattac<br>tgtgtgctcatcctttccattgccgaagtatagaatcactggtgtgcagaagatccaatgctcccagcct<br>atattgttctcaccgaaagtgcctgcgtatattcatccaaggaagtatctcgtggaaacaccaccggtag<br>acgagactccggagccatcggcagagaaccaatccacagaggggacacctgaacaaccaccacttataac<br>cgaggatgagaccaggactagaacgcctgagccgatcatcatcgaagaggaagaagaggatagcataagt<br>ttgctgtcagatggcccgacccaccaggtgctgcaagtcgaggcagacattcacgggccgccctctgtat<br>ctagctcatcctggtccattcctcatgcatccgactttgatgtggacagtttatccatacttgacaccct<br>ggagggagctagcgtgaccagcggggcaacgtcagccgagactaactcttacttcgcaaagagtatggag<br>tttctggcgcgaccggtgcctgcgcctcgaacagtattcaggaaccctccacatcccgctccgcgcacaa<br>gaacaccgtcacttgcacccagcagggcctgctcgagaaccagcctagtttccaccccgccaggcgtgaa<br>tagggtgatcactagagaggagctcgaggcgcttaccccgtcacgcactcctagcaggtcggtctcgaga<br>accagcctggtctccaacccgccaggcgtaaatagggtgattacaagagaggagtttgaggcgttcgtag<br>cacaacaacaatgacggtttgatgcgggtgcatacatcttttcctccgacaccggtcaagggcatttaca<br>acaaaaatcagtaaggcaaacggtgctatccgaagtggtgttggagaggaccgaattggagatttcgtat<br>gccccgcgcctcgaccaagaaaaagaagaattactacgcaagaaattacagttaaatcccacacctgcta<br>acagaagcagataccagtccaggaaggtggagaacatgaaagccataacagctagacgtattctgcaagg<br>cctagggcattatttgaaggcagaaggaaaagtggagtgctaccgaaccctgcatcctgttcctttgtat<br>tcatctagtgtgaaccgtgccttttcaagccccaaggtcgcagtggaagcctgtaacgccatgttgaaag<br>agaactttccgactgtggcttcttactgtattattccagagtacgatgcctatttggacatggttgacgg<br>agcttcatgctgcttagacactgccagtttttgccctgcaaagctgcgcagctttccaaagaaacactcc<br>tatttggaacccacaatacgatcggcagtgccttcagcgatccagaacacgctccagaacgtcctggcag<br>ctgccacaaaaagaaattgcaatgtcacgcaaatgagagaattgcccgtattggattcggcggcctttaa<br>tgtggaatgcttcaagaaatatgcgtgtaataatgaatattgggaaacgtttaaagaaaaccccatcagg<br>cttactgaagaaaacgtggtaaattacattaccaaattaaaaggaccaaaagctgctgctctttttgcga<br>agacacataatttgaatatgttgcaggacataccaatggacaggtttgtaatggacttaaagagagacgt<br>gaaagtgactccaggaacaaaacatactgaagaacggcccaaggtacaggtgatccaggctgccgatccg<br>ctagcaacagcgtatctgtgcggaatccaccgagagctggttaggagattaaatgcggtcctgcttccga<br>acattcatacactgtttgatatgtcggctgaagactttgacgctattatagccgagcacttccagcctgg<br>ggattgtgttctggaaactgacatcgcgtcgtttgataaaagtgaggacgacgccatggctctgaccgcg<br>ttaatgattctggaagacttaggtgtggacgcagagctgttgacgctgattgaggcggctttcggcgaaa<br>tttcatcaatacatttgcccactaaaactaaatttaaattcggagccatgatgaaatctggaatgttcct<br>cacactgtttgtgaacacagtcattaacattgtaatcgcaagcagagtgttgagagaacggctaaccgga<br>tcaccatgtgcagcattcattggagatgacaatatcgtgaaaggagtcaaatcggacaaattaatggcag<br>acaggtgcgccacctggttgaatatggaagtcaagattatagatgctgtggtgggcgagaaagcgcctta<br>tttctgtggagggtttattttgtgtgactccgtgaccggcacagcgtgccgtgtggcagacccccctaaaa<br>aggctgtttaagcttggcaaacctctggcagcagacgatgaacatgatgatgacaggagaagggcattgc<br>atgaagagtcaacacgctggaaccgagtgggtattctttcagagctgtgcaaggcagtagaatcaaggta<br>tgaaaccgtaggaacttccatcatagttatggccatgactactctagctagcagtgttaaatcattcagc<br>tacctgagaggggcccctataactctctacggctaacctgaatggactacgacatagtctagtccgccaa<br>gATGTGTCCTCAGAAGCTAACCATCTCCTGGTTTGCCATCGTTTTGCTGGTGTCTCCACTCATGGCCATG<br>TGGGAGCTGGAGAAAGACGTTTATGTTGTAGAGGTGGACTGGACTCCCGATGCCCCTGGAGAAACAGTGA<br>ACCTCACCTGTGACACGCCTGAAGAAGATGACATCACCTGGACCTCAGACCAGAGACATGGAGTCATAGG<br>CTCTGGAAAGACCCTGACCATCACTGTCAAAGAGTTTCTAGATGCTGGCCAGTACACCTGCCACAAAGGA<br>GGCGAGACTCTGAGCCACTCACATCTGCTGCTCCACAAGAAGGAAAATGGAATTTGGTCCACTGAAATTT<br>TAAAAAATTTCAAAAACAAGACTTTCCTGAAGTGTGAAGCACCAAATTACTCCGGACGGTTCACGTGCTC<br>ATGGCTGGTGCAAAGAAACATGGACTTGAAGTTCAACATCAAGAGCAGTAGCAGTTCCCCTGACTCTCGG<br>GCAGTGACATGTGGAATGGCGTCTCTGTCTGCAGAGAAGGTCACACTGGACCAAAGGGACTATGAGAAGT<br>ATTCAGTGTCCTGCCAGGAGGATGTCACCTGCCCAACTGCCGAGGAGACCCTGCCCATTGAACTGGCGTT<br>GGAAGCACGGCAGCAGAATAAATATGAGAACTACAGCACCAGCTTCTTCATCAGGGACATCATCAAACCA

	GACCCGCCCAAGAACTTGCAGATGAAGCCTTTGAAGAACTCACAGGTGGAGGTCAGCTGGGAGTACCCTG<br>ACTCCTGGAGCACTCCCCATTCCTACTTCTCCCTCAAGTTCTTTGTTCGAATCCAGCGCAAGAAAGAAAA<br>GATGAAGGAGACAGAGGAGGGGTGTAACCAGAAAGGTGCGTTCCTCGTAGAGAAGACATCTACCGAAGTC<br>CAATGCAAAGGCGGGAATGTCTGCGTGCAAGCTCAGGATCGCTATTACAATTCCTCATGCAGCAAGTGGG<br>CATGTGTTCCCTGCAGGGTCCGATCCGGTGGCAGTGGGGGTGGGTCTGGAGGGGGCAGCGGAGGAGGCTC<br>CAGGGTCATTCCAGTCTCTGGACCTGCCAGGTGTCTTAGCCAGTCCCGAAACCTGCTGAAGACCACAGAT<br>GACATGGTGAAGACGGCCAGAGAAAAACTGAAACATTATTCCTGCACTGCTGAAGACATCGATCATGAAG<br>ACATCACACGGGACCAAACCAGCACATTGAAGACCTGTTTACCACTGGAACTACACAAGAACGAGAGTTG<br>CCTGGCTACTAGAGAGACTTCTTCCACAACAAGAGGGAGCTGCCTGCCCCCACAGAAGACGTCTTTGATG<br>ATGACCCTGTGCCTTGGTAGCATCTATGAGGACTTGAAGATGTACCAGACAGAGTTCCAGGCCATCAACG<br>CAGCACTTCAGAATCACAACCATCAGCAGATCATTCTAGACAAGGGCATGCTGGTGGCCATCGATGAGCT<br>GATGCAGTCTCTGAATCATAATGGCGAGACTCTGCGCCAGAAACCTCCTGTGGGAGAAGCAGACCCTTAC<br>AGAGTGAAAATGAAGCTCTGCATCCTGCTTCACGCCTTCAGCACCCGCGTCGTGACCATCAACAGGGTGA<br>TGGGCTATCTGAGCTCCGCCTGATAAggcgcgcccacccagcggccgcatacagcagcaattggcaagct<br>gcttacatagaactcgcggcgattggcatgccgccttaaatttttattttatttttcttttcttttccg<br>aatcggatttgtttttaatatttcaaaaaaaaaaaaaaaaaaaaaaaaaaaaaaaagaag<br>agctagggataacagggtaattgagcaaaaggccagcaaaaggccaggaaccgtaaaaaggccgcgttgc<br>tggcgtttttccataggctccgcccccctgacgagcatcacaaaaatcgacgctcaagtcagaggtggcg<br>aaacccgacaggactataaagataccaggcgtttccccctggaagctccctcgtgcgctctcctgttccg<br>accctgccgcttaccggatacctgtccgcctttctcccttcgggaagcgtggcgctttctcatagctcac<br>gctgtaggtatctcagttcggtgtaggtcgttcgctccaagctgggctgtgtgcacgaaccccccgttca<br>gcccgaccgctgcgccttatccggtaactatcgtcttgagtccaacccggtaagacacgacttatcgcca<br>ctggcagcagccactggtaacaggattagcagagcgaggtatgtaggcggtgctacagagttcttgaagt<br>ggtggcctaactacggctacactagaagaacagtatttggtatctgcgctctgctgaagccagttacctt<br>cggaaaaagagttggtagctcttgatccggcaaacaaaccaccgctggtagcggtggtttttttgtttgc<br>aagcagcagattacgcgcagaaaaaaaggatctcaagaagatcctttgatcttttctacggggtctgacg<br>ctcagtggaacgaaaactcacgttaagggattttggtcatgagattatcaaaaaggatcttcacctagat<br>ccttttaaattaaaaatgaagttttaaatcaatctaaagtatatatgagtaaacttggtctgacagttag<br>aaaaactcatcgagcatcaaatgaaactgcaatttattcatatcaggattatcaataccatatttttgaa<br>aaagccgtttctgtaatgaaggagaaaactcaccgaggcagttccataggatggcaagatcctggtatcg<br>gtctgcgattccgactcgtccaacatcaatacaacctattaatttcccctcgtcaaaaataaggttatca<br>agtgagaaatcaccatgagtgacgactgaatccggtgagaatggcaaaagtttatgcatttctttccaga<br>cttgttcaacaggccagccattacgctcgtcatcaaaatcactcgcatcaaccaaaccgttattcattcg<br>tgattgcgcctgagcgagacgaaatacgcgatcgctgttaaaaggacaattacaaacaggaatcgaatgc<br>aaccggcgcaggaacactgccagcgcatcaacaatattttcacctgaatcaggatattcttctaatacct<br>ggaatgctgttttcccagggatcgcagtggtgagtaaccatgcatcatcaggagtacggataaaatgctt<br>gatggtcggaagaggcataaattccgtcagccagtttagtctgaccatctcatctgtaacatcattggca<br>acgctacctttgccatgtttcagaaacaactctggcgcatcgggcttcccatacaatcgatagattgtcg<br>cacctgattgcccgacattatcgcgagcccatttatacccatataaatcagcatccatgttggaatttaa<br>tcgcggcctagagcaagacgtttcccgttgaatatggctcatactcttcctttttcaatattattgaagc<br>atttatcagggttattgtctcatgagcggatacatatttgaatgtatttagaaaaataaacaaataggggg<br>ttccgcgcacatttccccgaaaagtgccacctgacgtctaagaaaccattattatcatgacattaaccta<br>taaaaataggcgtatcacgaggccctttcgtctagggataacagggtaattaatacgactcactatag

srRNA human IL-12 sequence<br><br>(Human IL-12 sequence is in uppercase letters)	ataggcggcgcatgagagaagcccagaccaattacctacccaaaatggagaaagttcacgttgacatcga<br>ggaagacagcccattcctcagagctttgcagcggagcttcccgcagtttgaggtagaagccaagcaggtc<br>actgataatgaccatgctaatgccagagcgttttcgcatctggcttcaaaactgatcgaaacggaggtgg<br>acccatccgacacgatccttgacattggaagtgcgcccgcccgcagaatgtattctaagcacaagtatca<br>ttgtatctgtccgatgagatgtgcggaagatccggacagattgtataagtatgcaactaagctgaagaaa<br>aactgtaaggaaataactgataaggaattggacaagaaaatgaaggagctcgccgccgtcatgagcgacc<br>ctgacctggaaactgagactatgtgcctccacgacgacgagtcgtgtcgctacgaagggcaagtcgctgt<br>ttaccaggatgtatacgcggttgacggaccgacaagtctctatcaccaagccaataagggagttagagtc<br>gcctactggataggctttgacaccaccccttttatgtttaagaacttggctggagcatatccatcatact<br>ctaccaactgggccgacgaaaccgtgttaacggctcgtaacataggcctatgcagctctgacgttatgga<br>gcggtcacgtagagggatgtccattcttagaaagaagtatttgaaaccatccaacaatgttctattctct<br>gttggctcgaccatctaccacgagaagagggacttactgaggagctggcacctgccgtctgtatttcact<br>tacgtggcaagcaaaattacacatgtcggtgtgagactatagttagttgcgacgggtacgtcgttaaaag<br>aatagctatcagtccaggcctgtatgggaagccttcaggctatgctgctacgatgcaccgcgagggattc<br>ttgtgctgcaaagtgacagacacattgaacggggagagggtctcttttcccgtgtgcacgtatgtgccag<br>ctacattgtgtgaccaaatgactggcatactggcaacagatgtcagtgcggacgacgcgcaaaaactgct<br>ggttgggctcaaccagcgtatagtcgtcaacggtcgcacccagagaaacaccaataccatgaaaaattac<br>cttttgcccgtagtggcccaggcatttgctaggtgggcaaaggaatataaggaagatcaagaagatgaaa<br>ggccactaggactacgagatagacagttagtcatggggtgttgttgggcttttagaaggcacaagataac<br>atctatttataagcgcccggatacccaaaccatcatcaaagtgaacagcgatttccactcattcgtgctg<br>cccaggataggcagtaacacattggagatcgggctgagaacaagaatcaggaaaatgttagaggagcaca<br>aggagccgtcacctctcattaccgccgaggacgtacaagaagctaagtgcgcagccgatgaggctaagga<br>ggtgcgtgaagccgaggagttgcgcgcagctctaccacctttggcagctgatgttgaggagcccactctg<br>gaagccgatgtcgacttgatgttacaagaggctggggccggctcagtggagacacctcgtggcttgataa<br>aggttaccagctacgatggcgaggacaagatcggctcttacgctgtgctttctccgcaggctgtactcaa<br>gagtgaaaaattatcttgcatccaccctctcgctgaacaagtcatagtgataacacactctggccgaaaa<br>gggcgttatgccgtggaaccataccatggtaaagtagtggtgccagagggacatgcaatacccgtccagg<br>actttcaagctctgagtgaaagtgccaccattgtgtacaacgaacgtgagttcgtaaacaggtacctgca<br>ccatattgccacacatggaggagcgctgaacactgatgaagaatattacaaaactgtcaagcccagcgag<br>cacgacggcgaatacctgtacgacatcgacaggaaacagtgcgtcaagaaagaactagtcactgggctag<br>ggctcacaggcgagctggtggatcctcccttccatgaattcgcctacgagagtctgagaacacgaccagc<br>cgctccttaccaagtaccaaccataggggtgtatggcgtgccaggatcaggcaagtctggcatcattaaa<br>agcgcagtcaccaaaaaagatctagtggtgagcgccaagaaagaaaactgtgcagaaattataaggacg<br>tcaagaaaatgaaagggctggacgtcaatgccagaactgtggactcagtgctcttgaatggatgcaaaca<br>ccccgtagagaccctgtatattgacgaagcttttgcttgtcatgcaggtactctcagagcgctcatagcc<br>attataagacctaaaaaggcagtgctctgcggggatcccaaacagtgcggtttttttaacatgatgtgcc<br>tgaaagtgcattttaaccacgagatttgcacacaagtcttccacaaaagcatctctcgccgttgcactaa<br>atctgtgacttcggtcgtctcaaccttgttttacgacaaaaaaatgagaacgacgaatccgaaagagact<br>aagattgtgattgacactaccggcagtaccaaacctaagcaggacgatctcattctcacttgtttcagag<br>ggtgggtgaagcagttgcaaatagattacaaaggcaacgaaataatgacggcagctgcctctcaagggct<br>gacccgtaaaggtgtgtatgccgttcggtacaaggtgaatgaaaatcctctgtacgcacccacctcagaa<br>catgtgaacgtcctactgacccgcacggaggaccgcatcgtgtggaaaacactagccggcgacccatgga<br>taaaaacactgactgccaagtaccctgggaatttcactgccacgatagaggagtggcaagcagagcatga<br>tgccatcatgaggcacatcttggagagaccggaccctaccgacgtcttccagaataaggcaaacgtgtgt<br>tgggccaaggctttagtgccggtgctgaagaccgctggcatagacatgaccactgaacaatggaacactg<br>tggattattttgaaacggacaaagctcactcagcagagatagtattgaaccaactatgcgtgaggttctt<br>tggactcgatctggactccggtctattttctgcacccactgttccgttatccattaggaataatcactgg<br>gataactccccgtcgcctaacatgtacgggctgaataaagaagtggtccgtcagctctctcgcaggtacc<br>cacaactgcctcgggcagttgccactggaagagtctatgacatgaacactggtacactgcgcaattatga<br>tccgcgcataaacctagtacctgtaaacagaagactgcctcatgctttagtcctccaccataatgaacac<br>ccacagagtgacttttcttcattcgtcagcaaattgaagggcagaactgtcctggtggtcggggaaaagt<br>tgtccgtcccaggcaaaatggttgactggttgtcagaccggcctgaggctaccttcagagctcggctgga<br>tttaggcatcccaggtgatgtgcccaaatatgacataatatttgttaatgtgaggaccccatataaatac<br>catcactatcagcagtgtgaagaccatgccattaagcttagcatgttgaccaagaaagcttgtctgcatc<br>tgaatcccggcggaacctgtgtcagcataggttatggttacgctgacagggccagcgaaagcatcattgg<br>tgctatagcgcggcagttcaagttttcccgggtatgcaaaccgaaatcctcacttgaagagacggaagtt<br>ctgtttgtattcattgggtacgatcgcaaggcccgtacgcacaatccttacaagctttcatcaaccttga<br>ccaacatttatacaggttccagactccacgaagccggatgtgcaccctcatatcatgtggtgcgaggga<br>tattgccacggccaccgaaggagtgattataaatgctgctaacagcaaaggacaacctggcggaggggtg<br>tgcggagcgctgtataagaaattcccggaaagcttcgatttacagccgatcgaagtaggaaaagcgcgac<br>tggtcaaaggtgcagctaaacatatcattcatgccgtaggaccaaacttcaacaaagtttcggaggttga<br>aggtgacaaacagttggcagaggcttatgagtccatcgctaagattgtcaacgataacaattacaagtca<br>gtagcgattccactgttgtccaccggcatcttttccgggaacaaagatcgactaacccaatcattgaacc<br>atttgctgacagctttagacaccactgatgcagatgtagccatatactgcagggacaagaaatgggaaat<br>gactctcaaggaagcagtggctaggagagaagcagtggaggagatatgcatatccgacgactcttcagtg<br>acagaacctgatgcagagctggtgagggtgcatccgaagagttctttggctggaaggaagggctacagca<br>caagcgatggcaaaactttctcatatttggaagggaccaagtttcaccaggcggccaaggatatagcaga<br>aattaatgccatgtggcccgttgcaacggaggccaatgagcaggtatgcatgtatatcctcggagaaagc

atgagcagtattaggtcgaaatgccccgtcgaagagtcggaagcctccacaccacctagcacgctgcctt
gcttgtgcatccatgccatgactccagaaagagtacagcgcctaaaagcctcacgtccagaacaaattac
tgtgtgctcatcctttccattgccgaagtatagaatcactggtgtgcagaagatccaatgctcccagcct
atattgttctcaccgaaagtgcctgcgtatattcatccaaggaagtatctcgtggaaacaccaccggtag
acgagactccggagccatcggcagagaaccaatccacagaggggacacctgaacaaccaccacttataac
cgaggatgagaccaggactagaacgcctgagccgatcatcatcgaagaggaagaagaggatagcataagt
ttgctgtcagatggcccgacccaccaggtgctgcaagtcgaggcagacattcacgggccgccctctgtat
ctagctcatcctggtccattcctcatgcatccgactttgatgtggacagtttatccatacttgacaccct
ggagggagctagcgtgaccagcggggcaacgtcagccgagactaactcttacttcgcaaagagtatggag
tttctggcgcgaccggtgcctgcgcctcgaacagtattcaggaaccctccacatcccgctccgcgcacaa
gaacaccgtcacttgcacccagcagggcctgctcgagaaccagcctagtttccaccccgccaggcgtgaa
tagggtgatcactagagaggagctcgaggcgcttaccccgtcacgcactcctagcaggtcggtctcgaga
accagcctggtctccaacccgccaggcgtaaatagggtgattacaagagaggagtttgaggcgttcgtag
cacaacaacaatgacggtttgatgcgggtgcatacatcttttcctccgacaccggtcaagggcatttaca
acaaaaatcagtaaggcaaacggtgctatccgaagtggtgttggagaggaccgaattggagatttcgtat
gccccgcgcctcgaccaagaaaaagaagaattactacgcaagaaattacagttaaatcccacacctgcta
acagaagcagataccagtccaggaaggtggagaacatgaaagccataacagctagacgtattctgcaagg
cctagggcattatttgaaggcagaaggaaaagtggagtgctaccgaaccctgcatcctgttcctttgtat
tcatctagtgtgaaccgtgccttttcaagccccaaggtcgcagtggaagcctgtaacgccatgttgaaag
agaactttccgactgtggcttcttactgtattattccagagtacgatgcctatttggacatggttgacgg
agcttcatgctgcttagacactgccagtttttgccctgcaaagctgcgcagctttccaaagaaacactcc
tatttggaacccacaatacgatcggcagtgccttcagcgatccagaacacgctccagaacgtcctggcag
ctgccacaaaaagaaattgcaatgtcacgcaaatgagagaattgcccgtattggattcggcggcctttaa
tgtggaatgcttcaagaaatatgcgtgtaataatgaatattgggaaacgtttaaagaaaccccatcagg
cttactgaagaaaacgtggtaaattacattaccaaattaaaaggaccaaaagctgctgctctttttgcga
agacacataatttgaatatgttgcaggacataccaatggacaggtttgtaatggacttaaagagagacgt
gaaagtgactccaggaacaaaacatactgaagaacggcccaaggtacaggtgatccaggctgccgatccg
ctagcaacagcgtatctgtgcggaatccaccgagagctggttaggagattaaatgcggtcctgcttccga
acattcatacactgtttgatatgtcggctgaagactttgacgctattatagccgagcacttccagcctgg
ggattgtgttctggaaactgacatcgcgtcgtttgataaaagtgaggacgacgccatggctctgaccgcg
ttaatgattctggaagacttaggtgtggacgcagagctgttgacgctgattgaggcggctttcggcgaaa
tttcatcaatacatttgcccactaaaactaaatttaaattcggagccatgatgaaatctggaatgttcct
cacactgtttgtgaacacagtcattaacattgtaatcgcaagcagagtgttgagagaacggctaaccgga
tcaccatgtgcagcattcattggagatgacaatatcgtgaaaggagtcaaatcggacaaattaatggcag
acaggtgcgccacctggttgaatatggaagtcaagattatagatgctgtggtgggcgagaaagcgcctta
tttctgtggagggtttattttgtgtgactccgtgaccggcacagcgtgccgtgtggcagacccccctaaaa
aggctgtttaagcttggcaaacctctggcagcagacgatgaacatgatgatgacaggagaagggcattgc
atgaagagtcaacacgctggaaccgagtgggtattctttcagagctgtgcaaggcagtagaatcaaggta
tgaaaccgtaggaacttccatcatagttatggccatgactactctagctagcagtgttaaatcattcagc
tacctgagaggggcccctataactctctacggctaacctgaatggactacgacatagtctagtccgccaa
gATGTGCCACCAGCAGCTGGTCATCAGCTGGTTCAGCCTCGTTTTCCTCGCCTCGCCGCTGGTCGCCATA
TGGGAGCTCAAGAAGGACGTATACGTGGTGGAGCTGGACTGGTACCCCGACGCGCCGGGCGAGATGGTCG
TCCTGACGTGCGACACGCCGGAGGAGGACGGCATCACGTGGACGCTGGACCAGTCCAGCGAGGTCCTCGG
CTCCGGCAAGACGCTGACGATCCAGGTCAAGGAGTTCGGCGACGCGGGCCAGTACACGTGCCACAAGGGC
GGCGAGGTCCTGAGCCACTCCCTCCTCCTGCTACACAAGAAGGAGGACGGGATCTGGAGCACGGACATCC
TCAAGGACCAGAAGGAGCCGAAGAACAAGACCTTCCTGCGCTGCGAGGCGAAGAATTACTCGGGCCGGTT
CACGTGCTGGTGGCTCACCACGATCAGCACGGACCTGACGTTCTCGGTCAAGTCGTCGCGGGGCTCGTCG
GACCCCCAGGGGGTGACCTGCGGCGCGGCGACGCTGTCGGCGGAGCGGGTGCGGGGCGACAACAAGGAGT
ACGAGTACTCGGTCGAGTGCCAGGAGGACTCGGCGTGCCCGGCGGCGGAGGAGTCGCTGCCGATCGAGGT
GATGGTCGACGCGGTCCACAAGCTGAAGTACGAGAACTACACGTCGTCGTTCTTCATCCGGGACATCATC
AAGCCGGACCCGCCGAAGAACCTGCAGCTGAAGCCGCTGAAGAACTCGCGGCAGGTCGAGGTCTCGTGGG
AGTACCCGGACACGTGGTCGACGCCGCACTCGTACTTCTCGCTGACGTTCTGCGTCCAAGTGCAGGGCAA
GTCGAAGCGGGAGAAGAAGGACCGGGTGTTCACCGACAAGACGAGCGCGACGGTGATCTGCCGGAAGAAC
GCGTCGATCTCGGTGCGGGCGCAGGACCGGTACTACTCGTCGTCGTGGTCGGAGTGGGCGTCGGTGCCGT
GCAGCGGCGGAAGCGGCGGCGGCAGCGGCGGAGGCTCTGGCGGCGGCTCCCGGAACCTGCCGGTGGCGAC
GCCGGACCCGGGGATGTTCCCGTGCCTGCACCACAGCCAGAACCTGCTGCGGGCGGTGTCGAACATGCTG
CAGAAGGCGCGGCAGACGCTGGAGTTCTACCCGTGCACGAGCGAGGAGATCGACCACGAGGACATCACGA
AGGACAAGACCAGCACGGTGGAGGCGTGCCTGCCGCTGGAGCTGACGAAGAACGAGTCGTGCCTGAACTC
GAGGGAGACGTCGTTCATCACGAACGGGTCGTGCCTGGCGTCGCGGAAGACGTCGTTCATGATGGCGCTG
TGCCTGTCGTCGATCTACGAGGACCTGAAGATGTACCAGGTGGAGTTCAAGACGATGAACGCGAAGCTGC
TGATGGACCCGAAGCGGCAGATCTTCCTCGACCAGAACATGCTGGCGGTGATCGACGAGCTCATGCAGGC
GCTCAACTTCAACAGCGAGACGGTGCCGCAGAAGTCGTCGCTCGAGGAGCCGGACTTCTACAAGACGAAG
ATCAAGCTCTGCATCCTGCTGCACGCTTTCCGGATCCGGGCGGTGACGATCGACCGGGTGATGTCGTACC
TGAACGCTTCGTGATAAggcgcgcccacccagcggccgcatacagcagcaattggcaagctgcttacata
gaactcgcggcgattggcatgccgccttaaatttttattttatttttcttttcttttccgaatcggatt
ttgtttttaatatttcaaaaaaaaaaaaaaaaaaaaaaaaaaaaaaaaaaaaaaaaagaagagctaggga
taacagggtaattgagcaaaaggccagcaaaaggccaggaaccgtaaaaaggccgcgttgctggcgtttt
tccataggctccgcccccctgacgagcatcacaaaaatcgacgctcaagtcagaggtggcgaaacccgac

	aggactataaagataccaggcgtttccccctggaagctccctcgtgcgctctcctgttccgaccctgccg<br>cttaccggatacctgtccgcctttctcccttcgggaagcgtggcgctttctcatagctcacgctgtaggt<br>atctcagttcggtgtaggtcgttcgctccaagctgggctgtgtgcacgaaccccccgttcagcccgaccg<br>ctgcgccttatccggtaactatcgtcttgagtccaacccggtaagacacgacttatcgccactggcagca<br>gccactggtaacaggattagcagagcgaggtatgtaggcggtgctacagagttcttgaagtggtggccta<br>actacggctacactagaagaacagtatttggtatctgcgctctgctgaagccagttaccttcggaaaaag<br>agttggtagctcttgatccggcaaacaaaccaccgctggtagcggtggtttttttgtttgcaagcagcag<br>attacgcgcagaaaaaaaggatctcaagaagatcctttgatcttttctacggggtctgacgctcagtgga<br>acgaaaactcacgttaagggattttggtcatgagattatcaaaaaggatcttcacctagatccttttaaa<br>ttaaaaatgaagttttaaatcaatctaaagtatatatgagtaaacttggtctgacagttagaaaaactca<br>tcgagcatcaaatgaaactgcaatttattcatatcaggattatcaataccatatttttgaaaaagccgtt<br>tctgtaatgaaggagaaaactcaccgaggcagttccataggatggcaagatcctggtatcggtctgcgat<br>tccgactcgtccaacatcaatacaacctattaatttcccctcgtcaaaaataaggttatcaagtgagaaa<br>tcaccatgagtgacgactgaatccggtgagaatggcaaaagtttatgcatttctttccagacttgttcaa<br>caggccagccattacgctcgtcatcaaaatcactcgcatcaaccaaaccgttattcattcgtgattgcgc<br>ctgagcgagacgaaatacgcgatcgctgttaaaaggacaattacaaacaggaatcgaatgcaaccggcgc<br>aggaacactgccagcgcatcaacaatattttcacctgaatcaggatattcttctaatacctggaatgctg<br>ttttcccagggatcgcagtggtgagtaaccatgcatcatcaggagtacggataaaatgcttgatggtcgg<br>aagaggcataaattccgtcagccagtttagtctgaccatctcatctgtaacatcattggcaacgctacct<br>ttgccatgtttcagaaacaactctggcgcatcgggcttcccatacaatcgatagattgtcgcacctgatt<br>gcccgacattatcgcgagcccatttatacccatataaatcagcatccatgttggaatttaatcgcggcct<br>agagcaagacgtttcccgttgaatatggctcatactcttcctttttcaatattattgaagcatttatcag<br>ggttattgtctcatgagcggatacatatttgaatgtatttagaaaaataaacaaataggggttccgcgca<br>catttccccgaaaagtgccacctgacgtctaagaaaccattattatcatgacattaacctataaaaatag<br>gcgtatcacgaggccctttcgtctagggataacagggtaattaatacgactcactatag

Supplementary Table 2. The serum concentration of human IL-12p70 in Cynomolgus Macaques after intravenous administration of JCXH-211. Each cynomolgus monkey was intravenously administrated with 100 μg JCXH-211 encoding human IL-12 on Day 1, Day 8, and Day 15. n = 2. Blood was collected at the indicated timepoints, and the concentration of human IL-12p70 in monkey serum was assessed by ELISA.

**Cytokine**	**Monkey**	**Day(s) Relative to Start Date**						
		-1	1.08	1.25	1.5	2	4	8.08
IL-12p70	Male	5.82	3.72	731.56	3613.48	4947.52	2129.23	108.47
	Female	7.00	5.82	2356.72	8851.11	7595.49	2232.32	174.19

Supplementary Table 3. Clinical observation and pathology test in Cynomolgus Monkey.

Observation Type	Monkey	Day(s) Relative to Start Date						
		16	17	18	19	20	21	22
Clinical observation	Male	No abnormality seen	No abnormality seen	No abnormality seen	No abnormality seen	No abnormality seen	No abnormality seen	No abnormality seen
	Female	No abnormality seen	No abnormality seen	No abnormality seen	No abnormality seen	No abnormality seen	No abnormality seen	No abnormality seen

Observation Type	Monkey	Day(s) Relative to Start Date					
		1	2	8	9	15	18
Inject site observation	Male	No abnormality seen	No abnormality seen	No abnormality seen	No abnormality seen	No abnormality seen	No abnormality seen
	Female	No abnormality seen	No abnormality seen	No abnormality seen	No abnormality seen	No abnormality seen	No abnormality seen

Pathology test	Monkey	Day(s) Relative to Start Date					
		-6	-1	7	14	21	22
Body weight (kg)	Male	2.65	2.54	2.59	2.66	2.69	2.65
	Female	2.00	2.01	2.11	2.09	2.09	2.02

Pathology test	Monkey	Day(s) Relative to Start Date		
		-1 to 7	-1 to 14	-1 to 21
Body weight gain (kg)	Male	0.05	0.12	0.15
	Female	0.10	0.08	0.08

Pathology test	Monkey	Day(s) Relative to Start Date			
		-1	1	8	15
Body temperature (℃)	Male	38.60	38.30	38.70	38.80
	Female	38.50	38.50	38.80	38.90

Pathology test	Monkey	Day(s) Relative to Start Date										
		2 to 3	3 to 4	4 to 5	5 to 6	6 to 7	7 to 8	8 to 9	9 to 10	10 to 11	11 to 12	12 to 13
Food mean daily consumption (g/animal/day)	Male	150.0	150.0	150.0	150.0	150.0	150.0	150.0	150.0	150.0	150.0	150.0
	Female	150.0	114.0	150.0	150.0	150.0	150.0	150.0	150.0	150.0	150.0	130.0

Pathology test	Monkey	Day(s) Relative to Start Date							
		13 to 14	14 to 15	15 to 16	16 to 17	17 to 18	18 to 19	19 to 20	20 to 21
Food mean daily consumption (g/animal/day)	Male	150.0	150.0	150.0	150.0	150.0	150.0	150.0	150.0
	Female	150.0	150.0	150.0	150.0	150.0	150.0	150.0	150.0

Pathology test	Monkey	Day(s) Relative to Start Date	Hematology									
			WBC (10^9/L)	Neut (10^9/L)	Neut (%)	Lymph (10^9/L)	Lymph (%)	Mono (10^9/L)	Mono (%)	Eos (10^9/L)	Eos (%)	Baso (10^9/L)
Hematology	Male	-1	15.80	6.48	41.00	8.28	52.40	0.71	4.50	0.12	0.80	0.10
		2	12.30	5.82	47.40	5.56	45.20	0.73	6.00	0.02	0.20	0.07
		8	20.63	7.27	35.20	11.14	54.00	0.96	4.70	0.14	0.70	0.16
		9	17.10	4.45	26.00	10.70	62.60	0.73	4.30	0.18	1.00	0.21
		22	16.32	4.78	29.30	10.22	62.60	0.76	4.70	0.27	1.70	0.15
	Female	-1	10.68	4.88	45.70	5.26	49.30	0.33	3.10	0.10	0.90	0.05
		2	7.80	4.25	54.50	3.12	40.00	0.37	4.80	0.00	0.00	0.02
		8	13.46	2.96	22.00	9.31	69.20	0.56	4.20	0.11	0.80	0.13
		9	13.70	2.09	15.30	10.38	75.80	0.51	3.70	0.04	0.30	0.14
		22	11.49	5.51	47.90	5.26	45.70	0.48	4.10	0.14	1.20	0.05

Pathology test	Monkey	Day(s) Relative to Start Date	Hematology									
			Baso (%)	RBC (10^12/L	HGB (g/L)	HCT (%)	Retic (10^12/L)	Retic (%)	MCV (fL)	MCHC (g/L)	MCH (pg)	PLT (10^9/L)
Hematology	Male	-1	0.70	5.70	125.00	41.20	0.09	1.58	72.20	304.00	21.90	491.00
		2	0.60	5.36	118.00	40.60	0.09	1.58	75.80	290.00	22.00	374.00
		8	0.80	4.30	94.00	34.10	0.03	0.78	79.50	274.00	21.80	513.00
		9	1.30	4.20	92.00	34.30	0.03	0.76	81.70	268.00	21.90	565.00
		22	0.90	4.73	103.00	39.90	0.17	3.52	84.30	258.00	21.70	521.00
	Female	-1	0.50	5.20	130.00	41.80	0.07	1.32	80.50	311.00	25.00	494.00
		2	0.30	4.90	121.00	39.20	0.05	0.92	80.20	308.00	24.70	424.00
		8	1.00	4.74	114.00	38.80	0.13	2.82	81.90	294.00	24.10	441.00
		9	1.00	4.35	106.00	36.30	0.14	3.32	83.50	292.00	24.40	447.00
		22	0.50	4.50	100.00	36.00	0.13	2.84	79.90	278.00	22.20	668.00

Pathology test	Monkey	Day(s) Relative to Start Date	Coagulation			Coagulation（CS5100）	
			PT (Seconds)	APTT (Seconds)	FIB (g/L)	P-FDP (μg/mL)	D-Dimer (mg/L)
Coagulation	Male	-1	9.50	20.00	1.69	1.10	0.19
		2	9.70	21.90	2.22	1.90	0.19
		8	9.30	24.30	6.06	20.80	0.19
		9	8.90	20.80	5.10	17.50	0.19
		22	8.00	18.50	1.71	2.00	0.19
	Female	-1	9.40	17.40	1.27	1.70	0.19
		2	10.50	23.60	1.58	1.50	0.19
		8	8.70	18.20	1.20	0.80	0.19
		9	9.00	19.80	1.18	4.00	0.19
		22	8.50	17.40	1.48	1.20	0.19

Pathology test	Monkey	Day(s) Relative to Start Date	CRP(mg/L)
CRP	Male	-1	6.10
		1	12.90
		8	46.60
		15	7.20
	Female	-1	6.70
		1	7.00
		8	11.80
		15	5.80

Pathology test	Monkey	Day(s) Relative to Start Date	Electrocardiography				
			Heart rate (HR) (/Min)	P-R interval (Seconds)	QRS duration (Seconds)	Q-T interval (Seconds)	Corrected QT Interval (QTc) (Seconds)
Electrocardio-graphy	Male	-1	171.00	0.07	0.03	0.16	0.27
		1	216.00	0.08	0.03	0.17	0.32
		8	277.00	0.07	0.03	0.13	0.28
		15	243.00	0.07	0.03	0.15	0.30
	Female	-1	211.00	0.06	0.03	0.16	0.30
		1	223.00	0.06	0.03	0.13	0.25
		8	252.00	0.05	0.03	0.14	0.29
		15	212.00	0.05	0.03	0.14	0.26

Pathology test	Monkey	Day(s) Relative to Start Date	Clinical Chemistry									
			ALT (U/L)	AST (U/L)	ALP (U/L)	TBil (μmol/L)	GGT (U/L)	TP (g/L)	Alb (g/L)	A/G	Glu (mmol/L)	UREA (mmol/L)
Chemistry	Male	-1	46.00	43.00	663.00	2.22	84.00	64.10	41.00	1.80	3.78	6.70
		2	49.00	44.00	367.00	1.99	45.00	69.40	39.10	1.30	4.15	5.50
		8	25.00	45.00	499.00	1.91	35.00	69.00	26.70	0.60	9.97	7.00
		9	30.00	50.00	468.00	1.43	38.00	73.90	28.90	0.60	3.54	6.70
		22	39.00	41.00	402.00	1.43	42.00	73.50	37.90	1.10	4.08	5.80
	Female	-1	44.00	62.00	400.00	3.44	47.00	70.30	40.10	1.30	3.46	8.60
		2	54.00	49.00	635.00	2.48	79.00	64.70	40.00	1.60	4.34	6.10
		8	62.00	67.00	572.00	1.93	68.00	66.40	38.10	1.30	5.37	6.90
		9	74.00	96.00	641.00	1.89	65.00	65.70	38.00	1.40	2.66	6.80
		22	40.00	35.00	567.00	1.36	68.00	68.00	40.00	1.40	4.51	6.80

Pathology test	Monkey	Day(s) Relative to Start Date	Clinical Chemistry									
			Cre (μmol/L)	CK (U/L)	LDH (U/L)	CHO (mmol/L)	TG (mmol/L)	Ca (mmol/L)	P (mmol/L)	Na^+ (mmol/L)	K^+ (mmol/L)	Cl^- (mmol/L)
Chemistry	Male	-1	41.00	328.00	464.00	3.72	0.37	2.74	2.00	147.30	4.75	108.90
		2	43.00	296.00	473.00	2.93	0.30	2.55	1.50	148.60	4.81	108.80
		8	46.00	180.00	752.00	2.68	1.13	2.17	1.56	146.00	4.80	103.70
		9	46.00	153.00	1278.00	3.02	1.15	2.39	1.67	153.80	7.05	112.90
		22	47.00	309.00	508.00	3.60	0.35	2.61	1.83	152.20	5.33	109.60
	Female	-1	47.00	603.00	549.00	3.59	0.59	2.70	2.57	145.10	5.31	105.20
		2	47.00	256.00	386.00	3.30	0.33	2.58	1.37	146.40	4.06	110.30
		8	42.00	272.00	653.00	2.34	0.61	2.50	1.66	145.90	4.98	107.40
		9	46.00	522.00	816.00	2.71	0.51	2.23	1.50	150.10	4.72	109.50
		22	43.00	191.00	371.00	3.84	0.43	2.49	1.63	146.00	4.65	106.40

Supplementary Table 4. The list of abbreviations to Supplementary Table 3.

WBC	Total leukocyte count
Neut	Neutrophilic granulocyte
Lymph	Lymphocyte
Mono	Mononuclear cell
Eos	Eosinophils
Baso	Basophils
RBC	Erythrocyte count
HGB	Hemoglobin
HCT	Hematocrit
Retic	Reticulocyte count
MCV	Mean corpuscular volume
MCH	Mean corpuscular hemoglobin
MCHC	Mean corpuscular hemoglobin concentration
PLT	Platelet
PT	Prothrombin time
APTT	Activated partial thromboplastin time
FIB	Fibrinogen
P-FDP	Plasma fibrin degradation products
CRP	C reaction protein
PR interval	The time from the onset of the P wave to the start of the QRS complex
QRS duration	Time from the start of the Q wave to the end of the S wave in one QRS comlex
Q-T interval	Time from the start of the Q wave to the end of the T wave
ALT	Alanine aminotransferase
AST	Aspartate aminotransferase
TP	Total protein
Alb	Albumin
A/G	Albumin/globulin ration
Tbil	Total bilirubin
LDH	Lactate dehydrogenase
ALP	Alkaline phosphatase
CK	Creatine kinase
GGT	γ-glutamyl transpeptidase
UREA	Urea
Cre	Creatinine
CHO	Total cholesterol
TG	Triglycerides
Glu	Glucose
Ca	Calcium
P	Phosphorus
Na^+	Sodium ion
K^+	Potassium ion
Cl^-	Chloride ion

Supplementary Table 5. Antibody list.

**Source**	**Antibody**	**Catalog number**
Biolegend	APC anti-mouse IFN-gamma	505810
Biolegend	PE anti-mouse TNF-α	506306
Biolegend	PE Rat IgG1, κ Isotype Ctrl	400408
Biolegend	APC Rat IgG1, κ Isotype	400412
Biolegend	Alexa Fluor® 700 Rat IgG2b, κ Isotype	400628
Biolegend	Brilliant Violet 711™ anti-mouse CD335 (NKp46)	137621
Biolegend	Brilliant Violet 421™ anti-mouse CD279 (PD-1)	135218
Biolegend	FITC anti-mouse CD45	103108
BioXcell	Anti-PD-1 (RMP1-14)	BP0146
Biolegend	PE/Cyanine5 anti-mouse CD3	100274
Biolegend	Brilliant Violet 605™ anti-mouse CD4	100451
Biolegend	Brilliant Violet 510™ anti-mouse CD8a	100752
Thermo Fisher Scientific	Live-dead AF780	65-0865-18
Biolegend	PerCP/Cyanine5.5 anti-mouse/human CD11b	101228
Biolegend	Brilliant Violet 605™ anti-mouse Ly-6C	128036
Biolegend	Brilliant Violet 510™ anti-mouse Ly-6G	127633
Thermo Fisher Scientific	Live/Dead Fixable Violet Dead Cell Stain Kit	L34964
Biolegend	Alexa Fluor 700 anti-human CD45	304024
Biolegend	Brilliant Violet 711 anti-human CD3	317328
Biolegend	APC/Cyanine7 anti-human CD8a	300926
BD Biosciences	BUV496 anti-human CD4	612936
Biolegend	PE/Dazzle 594 anti-human CD56	318348
Biolegend	PerCP anti-human HLA-DR	307628
Biolegend	Brilliant Violet 510 anti-human CD14	301842
BD Biosciences	APC anti-human CD19	555415
Biolegend	PE anti-human CD16	302008
Biolegend	Brilliant Violet 785™ anti-human CD279 (PD-1)	367432
Biolegend	Brilliant Violet 785™ mouse IgG1, κ isotype Ctrl	400170
Biolegend	Hu Fc Block Pure Fc1.3216 250ug	422302
